# Ranking and characterization of established BMI and lipid associated loci as candidates for gene-environment interactions

**DOI:** 10.1371/journal.pgen.1006812

**Published:** 2017-06-14

**Authors:** Dmitry Shungin, Wei Q. Deng, Tibor V. Varga, Jian'an Luan, Evelin Mihailov, Andres Metspalu, GIANT Consortium, Andrew P. Morris, Nita G. Forouhi, Cecilia Lindgren, Patrik K. E. Magnusson, Nancy L. Pedersen, Göran Hallmans, Audrey Y. Chu, Anne E. Justice, Mariaelisa Graff, Thomas W. Winkler, Lynda M. Rose, Claudia Langenberg, L. Adrienne Cupples, Paul M. Ridker, Nicholas J. Wareham, Ken K. Ong, Ruth J. F. Loos, Daniel I. Chasman, Erik Ingelsson, Tuomas O. Kilpeläinen, Robert A. Scott, Reedik Mägi, Guillaume Paré, Paul W. Franks

**Affiliations:** 1 Department of Clinical Sciences, Genetic & Molecular Epidemiology Unit, Lund University Diabetes Centre, Skåne University Hospital, Malmö, Sweden; 2 Department of Odontology, Umeå University, Umeå, Sweden; 3 Department of Public Health and Clinical Medicine, Unit of Medicine, Umeå University, Umeå, Sweden; 4 Broad Institute of the Massachusetts Institute of Technology and Harvard University, Cambridge, MA, United States of America; 5 Department of Statistical Sciences, University of Toronto, Toronto, Canada; 6 Novo Nordisk Foundation Center for Protein Research, Translational Disease Systems Biology Group, Faculty of Health and Medical Sciences, University of Copenhagen, Copenhagen, Denmark; 7 Department of Health Sciences, Exercise Physiology Group, Lund University, Lund, Sweden; 8 MRC Epidemiology Unit, University of Cambridge, Institute of Metabolic Science, Addenbrooke’s Hospital, Cambridge, United Kingdom; 9 Estonian Genome Center, University of Tartu, Tartu, Estonia; 10 Institute of Molecular and Cell Biology, University of Tartu, Tartu, Estonia; 11 Wellcome Trust Centre for Human Genetics, University of Oxford, Oxford, United Kingdom; 12 Department of Biostatistics, University of Liverpool, Liverpool, United Kingdom; 13 Department of Medical Epidemiology and Biostatistics, Karolinska Institutet, Stockholm, Sweden; 14 Department of Biobank Research, Umeå University, Umeå, Sweden; 15 Harvard Medical School, Boston, MA, United States of America; 16 Department of Epidemiology, University of North Carolina at Chapel Hill, Chapel Hill, NC, United States of America; 17 Department of Genetic Epidemiology, University of Regensburg, Regensburg, DE, Germany; 18 Division of Preventive Medicine, Brigham and Women's Hospital, Boston, MA, United States of America; 19 Department of Epidemiology and Public Health, UCL London, United Kingdom; 20 Department of Biostatistics, Boston University School of Public Health, Boston, MA; 21 The NHLBI Framingham Heart Study, Framingham, MA; 22 The Genetics of Obesity and Related Metabolic Traits Program, Icahn School of Medicine at Mount Sinai, New York, NY; 23 The Charles Bronfman Institute for Personalized Medicine, Icahn School of Medicine at Mount Sinai, New York, NY; 24 The Mindich Child Health and Development Institute, Icahn School of Medicine at Mount Sinai, New York, NY; 25 Science for Life Laboratory, Uppsala University, Uppsala, Sweden; 26 Department of Medical Sciences, Molecular Epidemiology, Uppsala University, Uppsala, Sweden; 27 Department of Medicine, Division of Cardiovascular Medicine, Stanford University School of Medicine, Stanford, CA, United States of America; 28 The Novo Nordisk Foundation Center for Basic Metabolic Research, Section of Metabolic Genetics, Faculty of Health and Medical Sciences, University of Copenhagen, Copenhagen, Denmark; 29 Department of Pathology and Molecular Medicine, McMaster University, Hamilton, Canada; 30 Department of Nutrition, Harvard TH Chan School of Public Health, Boston, Massachusetts, United States of America; 31 Oxford Centre for Diabetes, Endocrinology & Metabolism, Radcliff Department of Medicine, University of Oxford, Oxford, United Kingdom; University of Washington, UNITED STATES

## Abstract

Phenotypic variance heterogeneity across genotypes at a single nucleotide polymorphism (SNP) may reflect underlying gene-environment (G×E) or gene-gene interactions. We modeled variance heterogeneity for blood lipids and BMI in up to 44,211 participants and investigated relationships between variance effects (*P*_*v*_), G×E interaction effects (with smoking and physical activity), and marginal genetic effects (*P*_*m*_). Correlations between *P*_*v*_ and *P*_*m*_ were stronger for SNPs with established marginal effects (Spearman’s *ρ* = 0.401 for triglycerides, and *ρ* = 0.236 for BMI) compared to all SNPs. When *P*_*v*_ and *P*_*m*_ were compared for all pruned SNPs, only BMI was statistically significant (Spearman’s *ρ* = 0.010). Overall, SNPs with established marginal effects were overrepresented in the nominally significant part of the *P*_*v*_ distribution (*P*_*binomial*_ <0.05). SNPs from the top 1% of the *P*_*m*_ distribution for BMI had more significant *P*_*v*_ values (*P*_*Mann–Whitney*_ *=* 1.46×10^−5^), and the odds ratio of SNPs with nominally significant (<0.05) *P*_*m*_ and *P*_*v*_ was 1.33 (95% CI: 1.12, 1.57) for BMI. Moreover, BMI SNPs with nominally significant G×E interaction *P*-values (*P*_*int*_<0.05) were enriched with nominally significant *P*_*v*_ values (*P*_*binomial*_ = 8.63×10^−9^ and 8.52×10^−7^ for SNP × smoking and SNP × physical activity, respectively). We conclude that some loci with strong marginal effects may be good candidates for G×E, and variance-based prioritization can be used to identify them.

## Author summary

Most contemporary studies of gene-environment interactions focus on gene variants that are known to bear strong and reliable associations with the traits of interest. The strategy is intuitive because it helps limit the number of tests performed by focusing on a relatively small number of gene variants. However, this approach is predicated on an implicit assumption that these loci are strong candidates for interactions owing to their established relationships with the index traits. The counter-argument is that, because these loci have highly consistent signals within and between populations that vary by environmental characteristics, the probability that these variants interact with other factors is low. The current analysis tests whether variants with strong marginal effects signals (i.e., those prioritized through conventional genome-wide association analyses) are strong or weak candidates for gene-environment interactions. Here we describe analyses focused on lipids and BMI that test this hypothesis by comparing marginal effect signals with variance effect signals and those derived from explicit genome-wide, gene-environment interaction analyses. We conclude that for BMI, there are features of the top-ranking marginal effect loci that render them stronger candidates for interactions than is true of variants with weaker marginal effects signals. These findings are likely to help optimize the efficiency of future gene-environment interaction analyses by providing evidence-based rankings for strong candidate loci.

## Introduction

Gene-environment (G×E) interactions may contribute to complex diseases, but their detection has proven challenging; hence, a variety of approaches have been developed to enhance power. Most G×E analyses focus on loci that are strong biological candidates [1] or those with highly significant marginal effects [2]. The latter approach is attractive because these loci are available in many large cohorts, and can be conveniently followed-up with interaction analyses if environmental data are accessible. Moreover, selecting SNPs with strong and reproducible marginal effect signals is a pragmatic data-reduction step that may improve power [3], although this approach risks omitting other promising candidates [4].

In a linear regression setting, the presence of interaction effects drives phenotypic variance heterogeneity by genotype [3,5]. Exploiting variance heterogeneity as a signature of interactions is appealing because, unlike standard approaches for assessing G×E interactions, no explicit information about environmental exposures is needed [6] and multiple exposures can be simultaneously considered.

Here we explored whether loci identified in large-scale genome-wide association studies (GWAS) of blood lipids and body mass index (BMI) are strong candidates for G×E interactions by comparing genome-wide variance heterogeneity *P*-value distributions generated using Levene’s test against *P-*value distributions for marginal effects and explicit G×E interaction effects (for smoking and physical activity).

## Results

We assessed between-genotype variance heterogeneity for up to 1,927,671 directly genotyped or imputed SNPs (HapMap II CEU reference panel [7]) that passed quality control (QC). Meta-analyses of Levene’s test summary statistics [8] were performed for BMI (*n*≤44,211 participants), and blood concentrations of high-density lipoprotein cholesterol (HDL-C) (*n*≤34,315), low-density lipoprotein cholesterol (LDL-C) (*n*≤34,180), total cholesterol (TC) (*n*≤34,318) and triglycerides (TG) (*n*≤34,110). We then obtained marginal effects results for the same index traits and SNPs from publicly available GWAS summary data from the GIANT (Genetic Investigation of ANthropometric Traits) Consortium [9] and GLGC (Global Lipids Genetics Consortium) [10,11].

We compared the genome-wide marginal effects with between-genotype variance heterogeneity results for each of the five cardiometabolic traits by calculating the association between marginal effects (*P*_m_) and variance heterogeneity *(P*_v_*) P*-values using the rank-based Spearman correlation (*ρ*). This was done using a set of 42,710 pruned SNPs produced using the--*indep-pairwise* command in PLINK (see *Materials and Methods*) to account for linkage disequilibrium (LD) among variants.

**Fig 1:**
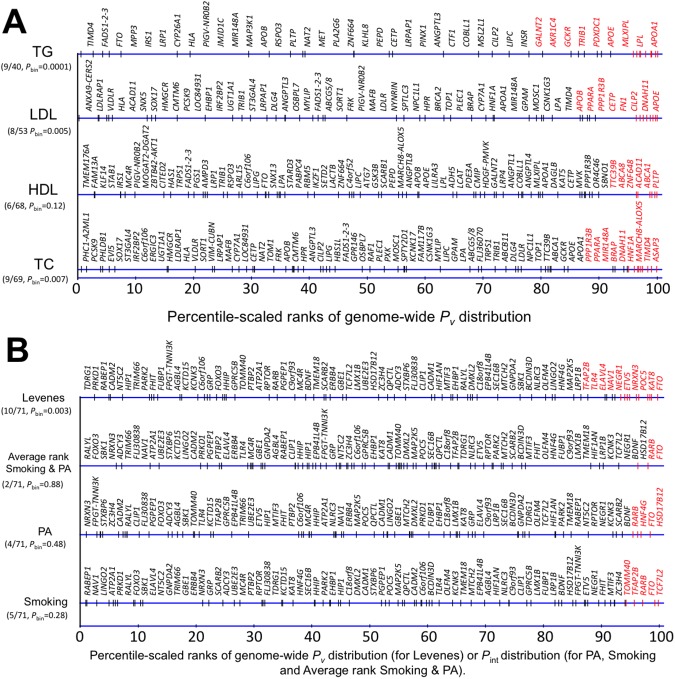
**A. Percentile-scaled ranks of GWAS-derived SNPs for lipid traits on the genome-wide distribution of *P*-values from Levene’s meta-analysis.** For each lipid trait (HDL-C, LDL-C, TG and TC on the vertical axis) we ranked *P*_v_ from Levene’s test for all SNPs from lowest to highest so that the lowest *P*_v_ for a given trait was assigned a rank equal to 1. We scaled ranks into percentiles such that the lowest *P*_v_ corresponded to the 100^th^ percentile. We then plotted percentile-scaled ranks of GWAS-derived loci (black sticks on the blue axis) on the distribution of percentile-scaled ranks of genome-wide *P*_v_ (blue axis) for each trait and marked in red loci with *P*_v_<0.05. Loci names are presented above the axis for *P*_v_ distribution of a given trait and are positioned in the same order as percentile-scaled ranks of GWAS-derived loci, but are equally spaced to facilitate cross-trait comparison (loci names with Levene’s test *P*_v_<0.05 are highlighted in red). To the left of each axis we present counts of GWAS-derived loci with *P*_v_<0.05 and total number of GWAS-derived loci in the analysis separated by a dash, as well as the *P*-value for the binomial test (*P*_binomial_). **B. Percentile-scaled ranks of GWAS-derived SNPs for BMI on the genome-wide distribution of *P*-values obtained from Levene’s test (*P***_**v**_**) and between-strata difference test *P*-values (*P***_**int**_**) from the ‘SNP × Physical Activity’ and ‘SNP × Smoking’ interaction tests for BMI.** For each analysis, we ranked *P*-values for all SNPs from lowest to highest so that the lowest *P*-value for a given trait was assigned a rank equal to 1. We scaled ranks into percentiles such that the lowest *P*-value corresponded to the 100^th^ percentile. We then plotted percentile-scaled ranks of GWAS-derived loci (black sticks on the blue axis) on the distribution of percentile-scaled ranks of genome-wide *P*-values (blue axis) from all four approaches and marked in red loci with *P*_v_<0.05 or *P*_int_<0.05 (or 95^th^ percentile for average rank between SNP **×** PA and SNP **×** Smoking). Loci names are presented above the axis for the *P*-value distribution of a given trait and are positioned in the same order as the percentile-scaled ranks of GWAS-derived loci, but are equally spaced to facilitate cross-trait comparisons (loci names with *P*_v_<0.05 or *P*_int_<0.05 are highlighted in red). To the left of each axis conveying each respective *P*-value distribution, we present counts of GWAS-derived BMI loci with *P*_v_<0.05 or *P*_int_<0.05 (or 95^th^ percentile for the average rank of the SNP **×** PA and SNP **×** Smoking interaction tests) and the total number of GWAS-derived loci in the analysis separated by a dash, as well as the *P*-value for the binomial test (*P*_binomial_).

As shown in Table 1 (see also Fig 1A and S1 Table), the Spearman’s *ρ* for the association between *P*_m_ and *P*_v_ for all pruned SNPs was of very small magnitude and only statistically significant for BMI. The exclusion of SNPs based on progressively more conservative *P*_m_ thresholds (*P*_*m*_*<*0.05; *P*_*m*_<10^−4^; previously established loci with *P*_*m*_<5×10^−8^ in external datasets), saw corresponding improvements in the magnitude of these correlations, which were statistically significant for all traits except TC when focusing on previously established loci. The BMI correlation at the *P*_*m*_<0.05 threshold, as well as the test of equality with *ρ* for all SNPs, was statistically significant, suggesting concordance between marginal and variance signals at a nominal level of significance. The odds ratio (OR) for a SNP to have both *P*_*m*_<0.05 and *P*_*v*_<0.05 as compared to *P*_*v*_≥0.05 was 1.33 (95% CI: 1.12, 1.57) for BMI while the 95% CIs of ORs for other traits included 1. On the other hand, the *P*-value for a non-zero *ρ* for TG was statistically significant when focusing on the established loci and at *P*_m_<10^−4^, suggesting concordance between marginal and variance signals at more conservative *P*_*m*_ thresholds.

**Table 1:** Spearman correlations between marginal effects *P*_m_ and heterogeneity of variance from Levene's test *P*_v_.

Trait	Max Sample Size	All SNPs in analysis			SNPs with *P*_m_<0.05				SNPs with *P*_m_<10^−4^				Known Loci				Odds ratio (SNPs with *P*_*m*_<0.05 and *P*_*v*_<0.05)
		# SNPs	Spearman *ρ*	*P*-value	# SNPs	Spearman *ρ*	*P*-value	*P*-value for equality test with *ρ* for all SNPs	# SNPs	Spearman *ρ*	*P*-value	*P*-value for equality test with *ρ* for all SNPs	# SNPs	Spearman *ρ*	*P*-value	*P*-value for equality test with *ρ* for all SNPs	OR (95% CI)
TC	34 318	41 328	0.001	0.89	2190	0.026	0.22	0.24	126	0.062	0.49	0.50	69	0.188	0.12	0.13	0.97 (0.78–1.19)
TG	34 110	41 206	0.003	0.51	2 079	-0.006	0.80	0.69	83	0.230	**3.61×10**^**−2**^	**3.87×10**^**−2**^	40	0.401	**1.03×10**^**−2**^	**1.00×10**^**−2**^	1.20 (0.99–1.44)
HDL-C	34 315	41 332	0.006	0.24	2 146	-0.001	0.97	0.77	95	-0.074	0.48	0.45	68	0.200	0.10	9.54×10^−2^	1.12 (0.92–1.35)
LDL-C	34 180	41 207	0.005	0.29	2 164	0.013	0.55	0.73	100	0.055	0.59	0.62	53	0.258	6.18×10^−2^	6.58×10^−2^	1.06 (0.87–1.28)
BMI	44 211	42 710	0.010	**4.56×10**^**−2**^	1 900	0.066	**3.82×10**^**−3**^	**1.56×10**^**−2**^	68	0.201	9.98×10^−2^	0.12	71	0.236	**4.76×10**^**−2**^	6.38×10^−2^	1.33 (1.12–1.57)

BMI: body mass index; HDL-C: low-density lipoprotein cholesterol; LDL-C: low-density lipoprotein cholesterol; SNP: single nucleotide polymorphism; TC: total cholesterol; TG: triglycerides

We further compared *P*_m_ with interaction *P*-values from exposure-specific (smoking and physical activity) genome-wide interaction tests for BMI (*P*_*int*_); this was only done for BMI owing to the requirement for an adequately powered external dataset (such a dataset was accessible through the GIANT consortium) (Table 2). Marginal effects GWAS were performed by strata of smokers vs. non-smokers and physically active vs. inactive participants (*n* = 210,316 European-ancestry adults [12]) respectively, and a heterogeneity test [12] was used to generate exposure specific *P*_*int*_ distributions. Spearman *ρ* for the pruned set of SNPs in the SNP ***×*** physical activity and the SNP ***×*** smoking analyses were low and not statistically significant (Table 2). We also compared *P*_*int*_ values and *P*_*v*_ values for BMI. Spearman’s *ρ* for the pruned set of SNPs were low and not statistically significant.

**Table 2:** Spearman correlations between *P*_*int*_ in *SNP × Physical Activity* and *SNP × Smoking* on BMI analyses and marginal effects *P*_m_ or heterogeneity of variance from Levene's test *P*_*v*_.

Characteristic	Max Sample Size	Max Sample Size PA/Smoking	All SNPs			SNPs with *P*_m_<0.05			Known SNPs		
			# SNPs	Spearman *ρ*	*P*-value	# SNPs	Spearman *ρ*	*P*-value	# SNPs	Spearman *ρ*	*P*-value
Marginal effects *P*_m_											
PA ***×*** SNP	322,144	180,271	41838	0.001	0.761	2142	0.029	0.176	71	-0.003	0.978
Smoking ***×*** SNP	322,144	210,306	41371	-0.004	0.429	2351	0.010	0.619	71	0.205	0.0863
Levene's test for homogeneity of variance *P*_v_											
PA ***×*** SNP	44,211	180,271	41838	0.005	0.35	2142	-0.003	0.884	71	0.052	0.669
Smoking ***×*** SNP	44,211	210,306	41371	0.004	0.401	2351	-0.023	0.265	71	0.110	0.360

PA: physical activity; BMI: body mass index; SNP: single nucleotide polymorphism; *P*_*v*_: Variance (Levene’s) test *P*-value; *P*_*m*_: Marginal (linear regression) test *P*-value

We next tested if the number of previously established marginal effect SNPs (*P*_m_<5***×***10^−8^) that were also nominally significant (*P*_v_<0.05) for variance heterogeneity was greater than expected by chance (Tables 3 and 4, Fig 1). For 4 out of the 5 index traits, we observed enrichment at the lower end of the *P*_*v*_ distribution (*P*_v_<0.05) for the established GWAS-derived lead SNPs. Thus, the nominally significant regions of the *P*_*v*_ distributions were generally enriched for GWAS-derived loci.

**Table 3:** Enrichment of variance and gene *×* environment interaction nominally significant results with GWAS-derived loci.

Trait	Analysis	Total SNPs/ Observed SNPs with *P*<0.05 (Expected)	*P*_*binomial*_
BMI	Levene's	71/10 (3.6)	**3*×*10**^**−3**^
	*SNP* ***×*** *PA*	71/4 (3.6)	0.48
	*SNP* ***×*** *Smoking*	71/5 (3.6)	0.28
	Average for *SNP* ***×*** *PA* & *SNP* ***×*** *Smoking*	71/2 (3.6)	0.88
TG	Levene's	40/9 (2)	**1*×*10**^**−4**^
LDL-C	Levene's	53/8 (2.7)	**5*×*10**^**−3**^
HDL-C	Levene's	68/6 (3.4)	0.12
TC	Levene's	69/9 (3.5)	**7*×*10**^**−3**^

PA: physical activity; BMI: body mass index; GWAS: genome-wide association study; HDL-C: low-density lipoprotein cholesterol; LDL-C: low-density lipoprotein cholesterol; SNP: single nucleotide polymorphism; TC: total cholesterol; TG: triglycerides

**Table 4:** Enrichment of SNPs with nominally significant *P*_int_ for test of *SNP × Smoking* and *SNP × Physical Activity* interaction for BMI (*P*_int_<0.05) by SNPs with nominally significant Levene's test (*P*_*v*_<0.05).

Analysis	Total SNPs with *P*_*int*_<0.05/ Observed SNPs with *P*_*int*_<0.05 & *P*_*v*_<0.05 (Expected)	*P*_binomial_
*SNP* ***×*** *PA*	2142/159 (107.1)	8.52***×***10^−7^
*SNP* ***×*** *Smoking*	2351/182 (117.6)	8.63***×***10^−9^

BMI: body mass index; PA: physical activity; SNP: single nucleotide polymorphism; *P*_*v*_ = Variance (Levene’s) test *P*-value; *P*_*int*_ = G***×***E interaction (heterogeneity) test *P*-value; *P*_binomial_ = significance of observing *P*_*v*_<0.05 more than expected by chance

We also performed enrichment analyses to test if previously established marginal effects SNPs (*P*_m_<5***×***10^−8^) are enriched for nominally significant (*P*_*int*_<0.05) interactions in the *SNP* ***×*** *physical activity* or *SNP* ***×*** *Smoking* analyses, but no enrichment was observed (Table 3; Fig 1B). By contrast, for the physical activity and smoking interaction tests (using all pruned SNPs), the lower end of the *P*_*int*_ distribution (*P*_*int*_<0.05) was enriched with SNPs that were nominally significant in the Levene’s test analysis (*P*_v_<0.05) (Table 4). This enrichment translated into an OR of 1.08 (95% CI: 1.01, 1.14) for a SNP to have *P*_*int*_<0.05 given *P*_*v*_<0.05 vs. *P*_*v*_≥0.05 for *SNP* ***×*** *physical activity* interaction. The corresponding OR for the *SNP* ***×*** *smoking* interaction test was not significant (OR = 1.02; 95% CI: 0.96, 1.08).

Finally, in the pruned SNP-set we used the Mann–Whitney U test to probe for systematic differences in *P*_*v*_ and *P*_*m*_ ranks. *P*-values were ordered from least significant to most significant, and the lowest 100^th^ centile (i.e. the most significantly associated SNPs) was compared to the remaining 99^th^ percentile for each of the five traits. For BMI, SNPs in the lowest 100^th^ centile of the *P*_*m*_ distribution had markedly higher *P*_*v*_ ranks (i.e. more significant *P*_*v*_) than the remaining SNPs (*P*_Mann–Whitney_ = 1.46***×***10^−5^; Table 5). Even when excluding previously established lead SNPs (*P*_m_<5***×***10^−8^) for BMI (or SNPs +/-500kb proximal), SNPs from the lowest 100^th^ centile of the *P*_*m*_ rank-ordered distribution had higher *P*_*v*_ ranks than the remaining SNPs (*P*_Mann–Whitney_ = 4.30***×***10^−4^; Table 5). Conversely, no difference in *P*_*v*_ ranks was observed for SNPs from the lowest 100^th^ centile of the *P*_*m*_ rank-ordered distribution for the four blood lipid traits; this may reflect trait-specific G***×***E effects or differences in statistical power by trait. No differences in *P*_*v*_ ranks between SNPs from the lowest 99^th^ centile of the *P*_*m*_ rank-ordered distribution compared to SNPs from the 98^th^ to 1^st^ centiles of the distribution were observed for any trait (*P*_Mann–Whitney_>0.05; Table 5). Similarly, no difference in *P*_*m*_ ranks was observed for SNPs from the lowest 100^th^ centile of the *P*_*v*_ rank-ordered distribution for any traits (*P*_Mann–Whitney_>0.05; Table 6).

**Table 5:** Comparison of Levene's test *P*_v_ ranks from different centiles of the *P*_m_ rank-ordered distribution for the index traits.

Trait	Known SNPs	Min *P*_m_ from 100th centile	Max *P*_m_ from 100th centile	Median *P*_v_ rank for 100th centile	Median *P*_v_ rank for 99th-1st centiles	Mann-Whitney P-value	Min *P*_m_ from 99th centile	Max *P*_m_ from 99th centile	Median *P*_v_ rank for 99^th^ centile	Median *P*_v_ rank for 98th-1st centiles	Mann-Whitney *P*-value
BMI	Included	4.78***×***10^−91^	5.82×10^−3^	58.82	49.93	**1.46×10**^**−5**^	5.86×10^−3^	1.85×10^−2^	52.79	49.91	0.42
BMI	Excluded	3.59×10^−6^	8.56×10^−3^	55.78	49.95	**4.30×10**^**−4**^	8.73×10^−3^	2.18×10^−2^	52.60	49.93	0.36
HDL-C	Included	3.56×10^−573^	6.48×10^−3^	51.49	49.99	0.47	6.48×10^−3^	1.67×10^−2^	50.49	49.98	0.92
HDL-C	Excluded	6.68×10^−11^	9.94×10^−3^	51.45	49.99	0.77	9.95×10^−3^	2.09×10^−2^	51.06	49.98	0.47
LDL-C	Included	3.80×10^−143^	7.14×10^−3^	53.11	49.98	0.52	7.18×10^−3^	1.75×10^−2^	48.44	49.99	0.85
LDL-C	Excluded	2.03×10^−11^	9.88×10^−3^	53.42	49.97	0.38	9.90×10^−3^	2.09×10^−2^	48.37	49.99	1.00
TG	Included	2.23×10^−113^	8.18×10^−3^	53.73	49.98	0.32	8.19×10^−3^	1.92×10^−2^	52.42	49.95	0.63
TG	Excluded	1.00×10^−10^	1.06×10^−2^	51.27	49.99	0.64	1.06×10^−2^	2.21×10^−2^	53.23	49.95	0.41
TC	Included	1.41×10^−107^	5.85×10^−3^	52.03	49.98	0.32	5.87×10^−3^	1.49×10^−2^	51.21	49.97	0.62
TC	Excluded	3.11×10^−11^	9.14×10^−3^	49.43	50.01	0.66	9.15×10^−3^	1.91×10^−2^	50.12	50.01	0.93

BMI: body mass index; HDL-C: low-density lipoprotein cholesterol; LDL-C: low-density lipoprotein cholesterol; SNP: single nucleotide polymorphism; TC: total cholesterol; TG: triglycerides; *P*_*v*_: Variance (Levene’s) test *P*-value; *P*_*m*_: marginal (linear regression) test *P*-value

**Table 6:** Comparison of marginal effects *P*_m_ ranks from different centiles of the Levene's test *P*_v_ rank-ordered distribution for the index traits.

Trait	Known SNPs	Min *P*_v_ from 100th centile	Max *P*_v_ from 100th centile	Median *P*_m_ rank for 100th centile	Median *P*_m_ rank for 99th-1st centiles	Mann-Whitney *P*-value	Min *P*_v_ from 99th centile	Max *P*_v_ from 99th centile	Median *P*_m_ rank for 99^th^ centile	Median *P*_m_ rank for 98th-1st centiles	Mann-Whitney *P*-value
BMI	Included	2.95×10^−7^	6.31×10^−3^	51.28	49.53	0.51	6.33×10^−3^	1.30×10^−2^	53.57	49.53	0.13
BMI	Excluded	2.95×10^−7^	6.38×10^−3^	51.40	49.48	0.42	6.38×10^−3^	1.30×10^−2^	53.50	49.44	0.17
HDL-C	Included	2.04×10^−5^	9.44×10^−3^	46.28	50.04	0.52	9.45×10^−3^	1.90×10^−2^	53.06	50.01	0.44
HDL-C	Excluded	2.04×10^−5^	9.45×10^−3^	46.42	50.05	0.37	9.47×10^−3^	1.89×10^−2^	53.37	50.01	0.31
LDL-C	Included	1.06×10^−8^	9.12×10^−3^	52.96	49.98	0.19	9.15×10^−3^	1.88×10^−2^	50.78	49.96	0.99
LDL-C	Excluded	1.44×10^−5^	9.37×10^−3^	50.39	49.99	0.64	9.37×10^−3^	1.92×10^−2^	51.85	49.97	0.68
TG	Included	2.45×10^−6^	8.39×10^−3^	48.93	50.01	0.60	8.39×10^−3^	1.78×10^−2^	51.75	50.01	0.53
TG	Excluded	2.45×10^−6^	8.37×10^−3^	49.23	50.01	0.66	8.39×10^−3^	1.78×10^−2^	51.92	50.00	0.51
TC	Included	3.28×10^−5^	1.08×10^−2^	51.61	49.98	0.16	1.08×10^−2^	2.09×10^−2^	50.29	49.98	0.92
TC	Excluded	3.28×10^−5^	1.10×10^−2^	51.23	50.00	0.33	1.10×10^−2^	2.10×10^−2^	49.92	50.00	0.93

BMI: body mass index; HDL-C: low-density lipoprotein cholesterol; LDL-C: low-density lipoprotein cholesterol; SNP: single nucleotide polymorphism; TC: total cholesterol; TG: triglycerides; *P*_*v*_: Variance (Levene’s) test *P*-value; *P*_*m*_: marginal (linear regression) test *P*-value

To assess whether a trait with a non-normal distribution (e.g. BMI) or strong marginal associations could cause spurious association between the marginal and variance signals, we recapitulated the analysis pipeline (correlation analysis, enrichment analysis, comparisons of rank *P*_*m*_ and *P*_*v*_ values) in simulations described in the *Materials and Methods*. Careful assessment of results emanating from these simulations did not reveal evidence of type I error inflation caused by the non-normal distribution of an outcome trait nor strong marginal effects. For instance, we extracted correlation *P*-values of *P*_*m*_, *P*_*v*_ and *P*_*int*_ generated from 5,000 simulations. QQ-plots of the 5,000 correlation *P*-values, 2,500 binomial *P*-values, and 2,500 Mann-Whitney U test *P*-values revealed no inflation (S1A–S1C Fig, S2A and S2B Fig and S3A and S3B Fig, respectively). Repeating these analyses on subsets of SNPs with low *P*_*m*_ values did not materially change the results.

## Discussion

Collectively, our analyses highlight a few variants with genome-wide significant marginal effects that may be strong candidates for G***×***E interactions owing to their strong concurrent variance heterogeneity *P*-values. For BMI, such SNPs are also overrepresented in the nominally significant part of the *P*_*v*_ distribution. *FTO* is an excellent example, as it conveys strong marginal effects [13], exhibits high between-genotype heterogeneity here (Tables 2 and 3 and Fig 1B) and elsewhere [5], and reportedly interacts with physical activity, diet and other lifestyle exposures [2,14,15] and is associated with macronutrient intake [16,17].

Although variance heterogeneity tests are potentially powerful screening tools for G***×***E interactions, like most interaction tests, they may be bias prone. For example, apparent differences in phenotypic variances across genotypes may be caused by scaling, particularly when the phenotypic means also differ substantially [18], such that the per-genotype means and variances for index traits are correlated. However, where necessary we transformed variables, and the correlations between *P*_*m*_ and *P*_*v*_ were generally weak, excluding this as a likely source of bias. Using simulated data, we investigated whether the non-normal distribution of a trait can cause a spurious association between marginal and variance signals, which we show is highly improbable. Through further simulations, we assessed whether SNPs with large marginal effects inflate *P*_*v*_, but observed no inflation, indicating that large genetic marginal effects do not artificially inflate variance heterogeneity to a meaningful extent, and SNPs with low *P*_*m*_ and low *P*_*v*_-values are thus likely to be strong candidates for G***×***E interactions, at least in the case of BMI. It might also be that combining populations from ancestral (e.g., hunter-gatherers) and contemporary environments increases variance heterogeneity owing to diversity in population substructure rather than G***×***E interactions *per se* [19]. However, this seems unlikely here, as the cohorts examined are from Westernized European-ancestry populations.

There are several additional explanations for between-genotype variance heterogeneity, such as variance misclassification that can occur when the index variant is located within a haplotype containing rare functional variants that convey strong marginal effects [5]. Hence, although variance heterogeneity tests represent a useful data-reduction step, before conclusions are drawn about the presence or absence of G***×***E interactions, index variants should be validated by testing their interactions with explicit environmental exposures, as we did here with smoking and physical activity. However, genome-wide G***×***E interactions datasets are not comprised of functionally validated G***×***E interactions, as no such resource is currently available for human complex traits. This limitation inhibits the extent to which causal effects can be attributed to the top-ranking loci and their interactions with smoking or physical activity.

We conclude that the common approach of prioritizing loci with established genome-wide significant association signals without further discrimination for G***×***E interaction analyses might be useful, but the efficiency of such analyses could be substantially improved by focusing on variants with low *P*-values for both variance heterogeneity and marginal effects. We provide these rankings here to facilitate this approach.

## Materials and methods

**Fig 2:**
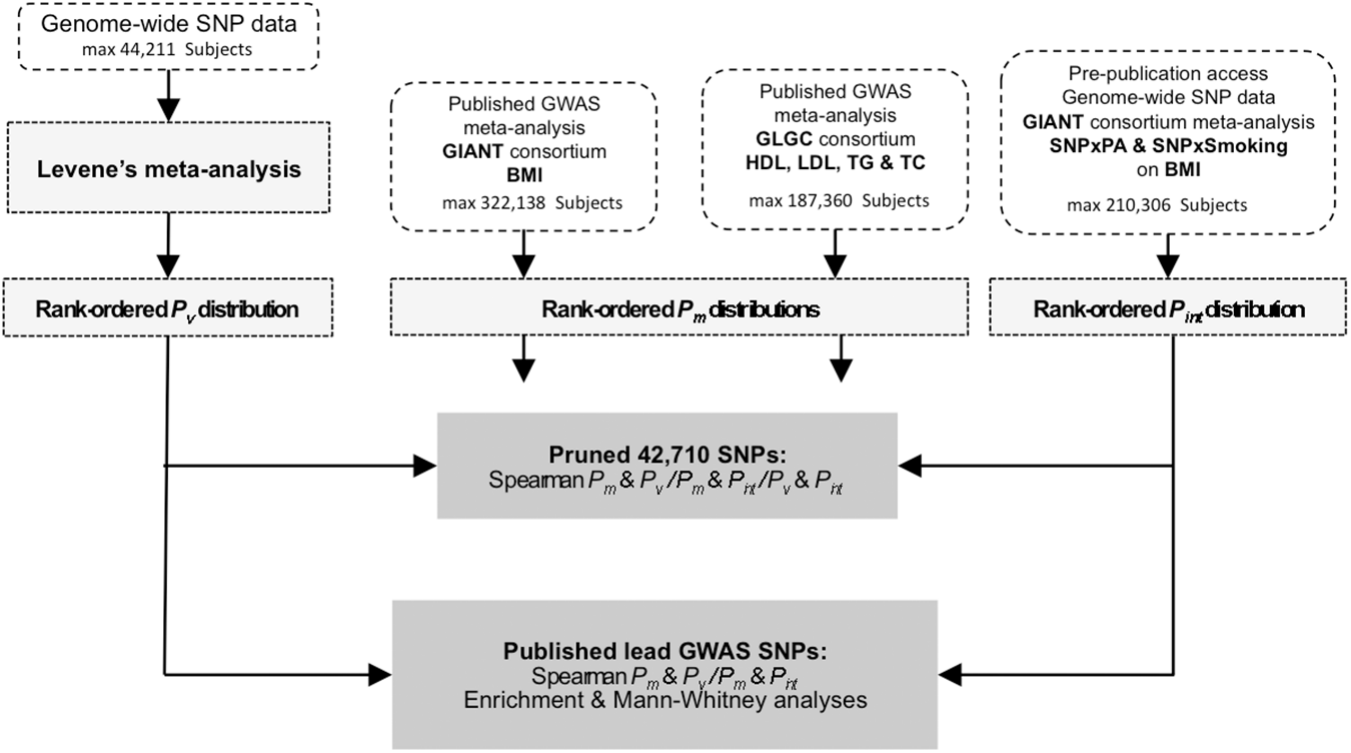
Data flow-chart. Three sources of genome-wide results were used: i) meta-analysis of Levene’s test results for between-genotype heterogeneity of phenotypic variances; ii) published results for marginal effects genome-wide association studies undertaken by the GIANT and GLGC consortia; iii) published results for SNP **×** physical activity and SNP **×** smoking in BMI (from the GIANT consortium).

A detailed project flow-chart is shown in Fig 2.

### Study sample

We performed a genome-wide search for SNPs whose associations with the following traits are characterized by high between-genotype variance heterogeneity: BMI, TC, TG, HDL-C and LDL-C. The variance heterogeneity analyses were performed using Levene’s test [20] in up to 44,211 participants of European descent from seven population-based cohorts. Descriptions of these cohorts are presented in S2 Table. To minimize bias that might result from unequal sample sizes between SNPs when calculating the correlations between the *P*-values from the marginal (*P*_*m*_) and variance heterogeneity (*P*_*v*_) meta-analyses, we restricted the sample size for analyses to 26,000 participants for BMI and to 24,000 participants for lipid traits (S4 Fig).

### Genotyping and imputation

A detailed summary of sample sizes, genotyping platforms, genotype calling algorithms, sample and SNP quality control filters, and analysis software for all participating cohorts are provided in S2 and S3 Tables. For each individual, SNPs were imputed using the CEU reference panel of HapMap II [7] (S2 Table). We excluded SNPs with low imputation quality (below 0.3 for MACH, 0.4 for IMPUTE, and 0.8 for PLINK imputed data), Hardy-Weinberg equilibrium *P* <10^−6^, directly genotyped SNP call rate < 95%, and minor allele frequency (MAF) < 1%.

### Selection of SNPs identified through GWAS

We identified SNPs that have been robustly associated (*P*<5x10^-8^) with the five cardiometabolic traits in European ancestry populations: 77 SNPs associated with BMI discovered by GIANT [9]; and 58 SNPs associated with LDL-C, 71 SNPs associated with HDL-C, 74 SNPs associated with TC, and 40 SNPs associated with TG [10,11] discovered by GLGC.

### Variance heterogeneity analyses

We used Levene’s test [20] to identify SNPs that show heterogeneity of phenotypic variances (*σ*_*i*_^2^) across the three genotype groups at each SNP locus (*i = 0*, *1*, *or 2)*. We first log_10_ transformed all five traits followed by a *z*-score transformation by subtracting the sample mean and dividing by the sample standard deviation (SD), and further Winsorized the *z*-score values at 4 SD. The transformed phenotype *Y* was then used to calculate *Z*, defined by the absolute deviation of each participant’s phenotype from the sample mean of his or her respective genotype group at a given SNP locus. For each trait, participating cohorts provided the necessary summary statistics for each genotype at each marker [8]. Specifically, the per genotype group counts (*n*_*0s*_, *n*_*1s*_, *n*_*2s*_), per genotype means (${\bar{Z}}_{0s},{\bar{Z}}_{1s},{\bar{Z}}_{2s}$), and per genotype group variances of *Z* (*σ*_0*s*_^2^,*σ*_1*s*_^2^,*σ*_2*s*_^2^) were centrally collected and meta-analyzed. The minimum number of observations per genotype group required is 30 participants per cohort.

Meta-analyses were performed using the following formula, derived previously [8]:

$$L=\frac{\left(N-3\right)}{\left(3-1\right)}\cdot \frac{(\sum_{i=0}^{2}\gamma_{i}\cdot {\left(\sum_{s}{\bar{Z}}_{is}\cdot \omega_{is}\right)}^{2}-(\sum_{i=0}^{2}\sum_{s}{\bar{Z}}_{is}\cdot \omega_{is}\cdot \gamma_{i}{)}^{2})}{\sum_{i=0}^{2}(\sum_{s}(\sigma_{Z_{is}}{}^{2}\cdot \omega_{is}-\frac{\sigma_{Z_{is}}{}^{2}}{N\cdot \gamma_{i}}+{\bar{Z}}_{is}{}^{2}\cdot \omega_{is})\cdot \gamma_{i}-({\left(\sum_{s}{\bar{Z}}_{is}\cdot \omega_{is}\right)}^{2}\cdot \gamma_{i}))}$$

Where *N* is the combined sample size, ${\bar{Z}}_{is}$ and $\sigma_{Z_{is}}{}^{2}$ are the sample mean and variance of *Z* in the *i*^*th*^ genotype group of the *s*^*th*^ study, respectively. When combining summary-level data to calculate the Levene’s test statistics *L*, the following natural weights *ω*_*is*_ and *γ*_*i*_ were calculated: $\omega_{is}=\frac{n_{is}}{\sum_{s}n_{is}}$ and $\gamma_{i}=\frac{n_{i}}{N}$, where *n*_*i*_ the sum of genotype counts in the *i*^*th*^ genotype group across all participating cohorts. These weights are determined by the frequency of the marker amongst the cohorts, such that the sum of both weights is equal to 1, i.e. $\sum_{s}\omega_{is}=1$ and $\sum_{i}\gamma_{i}=1$. The meta-analysis Levene’s test *P*-value is obtained by comparing *L* to an *F*-distribution with df_1_ = 2 and df_2_ = *N*-3.

### Comparison between marginal effects and variance heterogeneity P-values

Marginal effects *P*-values for BMI and the relevant lipid traits were obtained from publically available GWAS summary data from the GIANT [9] and GLGC [10,11] consortia, respectively (all cohorts included here in the Levene’s meta-analysis were also included in the GIANT and GLGC datasets).

To illustrate our findings, we rank-ordered the *P*-values (from lowest to highest) from both marginal effects and variance effects analyses for all 1,927,671 SNPs so that the lowest *P*-value for a given trait was assigned a rank equal to the lowest 100^th^ centile. These rank-scaled distributions for *P*_*m*_ for all five traits are presented in Fig 1.

We calculated Spearman’s correlations for each of the five cardiometabolic traits between *P*_*m*_ *and P*_*v*_. This was done using a pruned set of SNPs. Pruning was performed in the TwinGene cohort using the--*indep-pairwise 50 5 0*.*1* command in PLINK [21] by calculating LD (*r*^*2*^) for each pair of SNPs within a window of 50 SNPs, removing one of a pair of SNPs if *r*^*2*^>0.1; we proceeded by shifting the window 5 SNPs forwards and repeating the procedure. Spearman’s correlations were computed for categories of SNPs: i) all pruned SNPs, ii) the subset of SNPs that was nominally significant (*P*_*m*_<0.05) in the marginal effects analysis, iii) the subset of SNPs with *P*_*m*_<10^−4^ in the marginal effects analysis, and iv) SNPs that were previously established in conventional marginal effects GWAS meta-analyses (*P*_m_<5***×***10^−8^). We also compared Spearman’s correlations between these categories of SNPs using the test for equality of two correlations [22].

Next, we performed enrichment analyses to test if there was a higher number of established SNPs in the nominally significant variance *P-*value (*P*_*v*_<0.05) distribution than expected by chance under the binominal distribution.

We also tested if there is a difference in *P*_*v*_ ranks for SNPs from the lowest 100^th^ centile of the *P*_*m*_ rank-ordered distribution for all five traits and the rest of SNPs in the pruned set of SNPs using the Mann–Whitney U test, including and excluding established SNPs (or SNPs that were +/-500kb from the reported lead SNP). This analysis was repeated for SNPs from the 99^th^ centile vs SNPs from 1^st^ to 98^th^ centiles of the *P*_*m*_ rank-ordered distribution. The same Mann–Whitney U tests were used to study differences in *P*_*m*_ ranks for SNPs from the lowest 100^th^ and 99^th^ centiles of the *P*_*v*_ rank-ordered distribution and the rest of SNPs in the pruned set of SNPs.

All analyses were performed using Stata 12 (StataCorp LP, TX, USA), unless specified otherwise.

### SNP × Physical activity and SNP × Smoking interaction analyses for the outcome of BMI

We used now published data from 210,316 European-ancestry adults (from the GIANT consortium) pertaining to marginal effects meta-analyses for BMI that had been performed separately by strata of smoking (45,968 smokers vs. 164,355 non-smokers) [23]. The genetic marginal effect estimates, calculated separately within each of the two strata, were compared using a heterogeneity test [12] to infer the presence or absence of SNP ***×*** smoking interaction effects. The same analyses were performed using physical activity as a binary stratifying variable in up to 180,287 European-ancestry adults (42,065 physically active vs. 138,222 physically inactive) [24]. We calculated Spearman correlations between the *P*-values derived from the marginal effects meta-analysis and the *P*_int_ from the interaction effects meta-analysis (i.e., the between-strata heterogeneity test for *SNP* ***×*** *smoking* and *SNP* ***×*** *physical activity* interactions from the GIANT consortium); these tests were undertaken for all SNPs and those SNPs that were nominally significant (*P*_*m*_<0.05) in the marginal effects analysis. We then performed enrichment analyses to test if the numbers of nominally significant (*P*_int_<0.05) GWAS-derived SNPs from both *SNP* ***×*** *physical activity* and *SNP* ***×*** *smoking* analyses were greater than expected by chance under the binomial distribution. We further calculated the OR of having *P*_int_<0.05 given *P*_v_<0.05 versus *P*_v_≥0.05 both *SNP* ***×*** *physical activ*ity and *SNP* ***×*** *smoking* interaction analyses in a pruned set of TwinGene SNPs produced using the—*indep-pairwise 50 5 0*.*8* command in PLINK [21].

Thereafter, we calculated the average rank for each SNP’s ranking on the *P*_int_ rank-ordered distributions from the *SNP* ***×*** *smoking* and *SNP* ***×*** *physical activity* interaction analyses and performed enrichment analysis using these average ranks with >95^th^ centile instead of *P*_int_<0.05 as the cut-off.

### Simulations

We simulated genetic data for 44,000 individuals from a pruned set of 50,335 SNPs with allele frequencies, effect estimates and *P*_*m*_ values drawn from the GIANT consortium. We generated an outcome trait by summing the products of the simulated allele counts and effect estimates over all SNPs for each individual, and subsequently added a randomly generated non-normal error term such that the trait resembles the observed distribution of the transformed BMI trait used in the main (real data) analyses. We also simulated a fixed binary interacting factor with 30% prevalence. Using this simulated dataset, we calculated *P*_*m*_, *P*_*v*_ and *P*_*int*_ values for each SNP and undertook i) pairwise Spearman correlation analyses between *P*_*m*_, *P*_*v*_ and *P*_*int*_ values (5,000 simulations), ii) enrichment analysis using binomial tests (2,500 simulations) and iii) Mann-Whitney U tests to determine systematic differences in *P*_*v*_ and *P*_*m*_ ranks (2,500 simulations). Following the same pipeline, we created additional simulated datasets narrowing down SNPs to i) those with *P*_*m*_ values from the lowest percentile (*n* = 504; highest *P*_*m*_ = 5***×***10^−3^) and to ii) genome-wide significant SNPs (*n* = 71; *P*_*m*_<5***×***10^−8^), and tested the pairwise Spearman correlation for *P*_*m*_, *P*_*v*_ and *P*_*int*_ values (1,000 simulations for both sets). Simulations were run using the statistical software *R* (v. 3.3.2).[25]

## Supporting information

S1 Fig **A: Quantile-quantile plot of Spearman correlation test *P*-values for ranks of *P***_***m***_ **and *P***_***v***_. Quantile-quantile plot of Spearman correlation test *P*-values for ranks of *P*_*m*_ and *P*_*v*_. The figure illustrates 5,000 Spearman correlation *P* values testing for correlation between *P*_*m*_ and and *P*_*v*_ values drawn from a simulated dataset of 44,000 individuals and 50,335 SNPs. In the figure, distribution under the null hypothesis is represented as a black line while its 95% confidence interval is represented as dashed gray lines. The dashed red line represents the correlation P value obtained from the “real data” analysis presented in the main text. **B. Quantile-quantile plot of Spearman correlation test *P*-values for ranks of *P***_***m***_ **and *P***_***int***_. Quantile-quantile plot of Spearman correlation test *P*-values for ranks of *P*_*m*_ and *P*_*int*_. The figure illustrates 5,000 Spearman correlation *P* values testing for correlation between *P*_*m*_ and and *P*_*int*_ values drawn from a simulated dataset of 44,000 individuals and 50,335 SNPs. In the figure, distribution under the null hypothesis is represented as a black line while its 95% confidence interval is represented as dashed gray lines. **C. Quantile-quantile plot of Spearman correlation test *P*-values for ranks of *P***_***int***_ **and *P***_***v***_. Quantile-quantile plot of Spearman correlation test *P*-values for ranks of *P*_*int*_ and *P*_*v*_. The figure illustrates 5,000 Spearman correlation *P* values testing for correlation between *P*_*int*_ and and *P*_*v*_ values drawn from a simulated dataset of 44,000 individuals and 50,335 SNPs. In the figure, distribution under the null hypothesis is represented as a black line while its 95% confidence interval is represented as dashed gray lines.
(TIF)

S2 Fig **A. Quantile-quantile plot of binomial test *P*-values for enrichment of variants with *P***_***v***_**<0.05 among variants with *P***_***m***_**<0.05.** Quantile-quantile plot of binomial test *P*-values for enrichment of variants with *P*_*v*_<0.05 among variants with *P*_*m*_<0.05. The figure illustrates 2,500 binomial *P* values testing for enrichment of variants with *P*_*v*_<0.05 among all variants with *P*_*m*_<0.05. *P*_*v*_ and and *P*_*m*_ values drawn from a simulated dataset of 44,000 individuals and 50,335 SNPs. In the figure, distribution under the null hypothesis is represented as a black line while its 95% confidence interval is represented as dashed gray lines. **B. Quantile-quantile plot of binomial test *P*-values for enrichment of variants with *P***_***v***_**<0.05 among variants with *P***_***int***_**<0.05.** Quantile-quantile plot of binomial test *P*-values for enrichment of variants with *P*_*v*_<0.05 among variants with *P*_*int*_<0.05. The figure illustrates 2,500 binomial *P* values testing for enrichment of variants with *P*_*v*_<0.05 among all variants with *P*_*int*_<0.05. *P*_*v*_ and and *P*_*int*_ values drawn from a simulated dataset of 44,000 individuals and 50,335 SNPs. In the figure, the distribution under the null hypothesis is represented as a black line while its 95% confidence interval is represented as dashed gray lines. The dashed red line represents the correlation *P* value obtained from the “real data” analysis presented in the main text.
(TIF)

S3 Fig **A. Quantile-quantile plot of Mann-Whitney U test *P*-values for systematic differences in *P***_***v***_ **ranks among variants with top ranking and lower ranking *P***_***m***_ **values.** Quantile-quantile plot of Mann-Whitney U test *P*-values for systematic differences in *P*_*v*_ ranks among variants with top ranking and lower ranking *P*_*m*_ values. The figure illustrates 2,500 Mann-Whitney U *P* values testing for systematic differences in *P*_*v*_ ranks among those variants with the most significant *P*_*m*_ values (100^th^ percentile of *P*_*m*_ distribution) and the remaining variants (1–99 percentile of *P*_*m*_ distribution). *P*_*v*_ and and *P*_*m*_ values drawn from a simulated dataset of 44,000 individuals and 50,335 SNPs. In the figure, distribution under the null hypothesis is represented as a black line while its 95% confidence interval is represented as dashed gray lines. The dashed red line represents the correlation *P* value obtained from the “real data” analysis presented in the main text. **B. Quantile-quantile plot of Mann-Whitney U test *P*-values for systematic differences in *P***_***m***_ **ranks among variants with top ranking and lower ranking *P***_***v***_ **values.** Quantile-quantile plot of Mann-Whitney U test *P*-values for systematic differences in *P*_*m*_ ranks among variants with top ranking and lower ranking *P*_*v*_ values. The figure illustrates 2,500 Mann-Whitney U *P* values testing for systematic differences in *P*_*m*_ ranks among those variants with the most significant *P*_*v*_ values (100^th^ percentile of *P*_*v*_ distribution) and the remaining variants (1–99 percentile of *P*_*v*_ distribution). *P*_*v*_ and and *P*_*m*_ values drawn from a simulated dataset of 44,000 individuals and 50,335 SNPs. In the figure, distribution under the null hypothesis is represented as a black line while its 95% confidence interval is represented as dashed gray lines. The dashed red line represents the correlation *P* value obtained from the “real data” analysis presented in the main text.
(TIF)

S4 Fig Quantile-quantile plots of Levene’s test *P*-values for SNP associations with lipid traits and BMI. Associations between SNPs and BMI (A), LDL (B), HDL (C), TG (D), TC (E) are presented. Only SNPs with N ≥ 26,000 samples for BMI and N ≥ 24,000 for lipid traits are shown. In each sub-figure, distribution under the null hypothesis is represented as a black line while its 95% confidence interval is represented as dashed gray lines.
(TIF)

S1 Table Detailed results for known BMI, LDL-C, HDL-C, TG and TC loci. (XLSX)

S2 Table Study design, number of participants and sample quality control for genome-wide association study cohorts. (XLSX)

S3 Table Information on genotyping methods, quality control of SNPs, imputation, and statistical analysis. (XLSX)

S1 Text GIANT consortium contributors and their affiliations. (PDF)
