# Supplementary figures and images for: Ranking and characterization of established BMI and lipid associated loci as candidates for gene-environment interactions
Source: PLoS Genet. 2017 Jun 14;13(6):e1006812. doi: 10.1371/journal.pgen.1006812 (PMC5489225; doi:10.1371/journal.pgen.1006812)

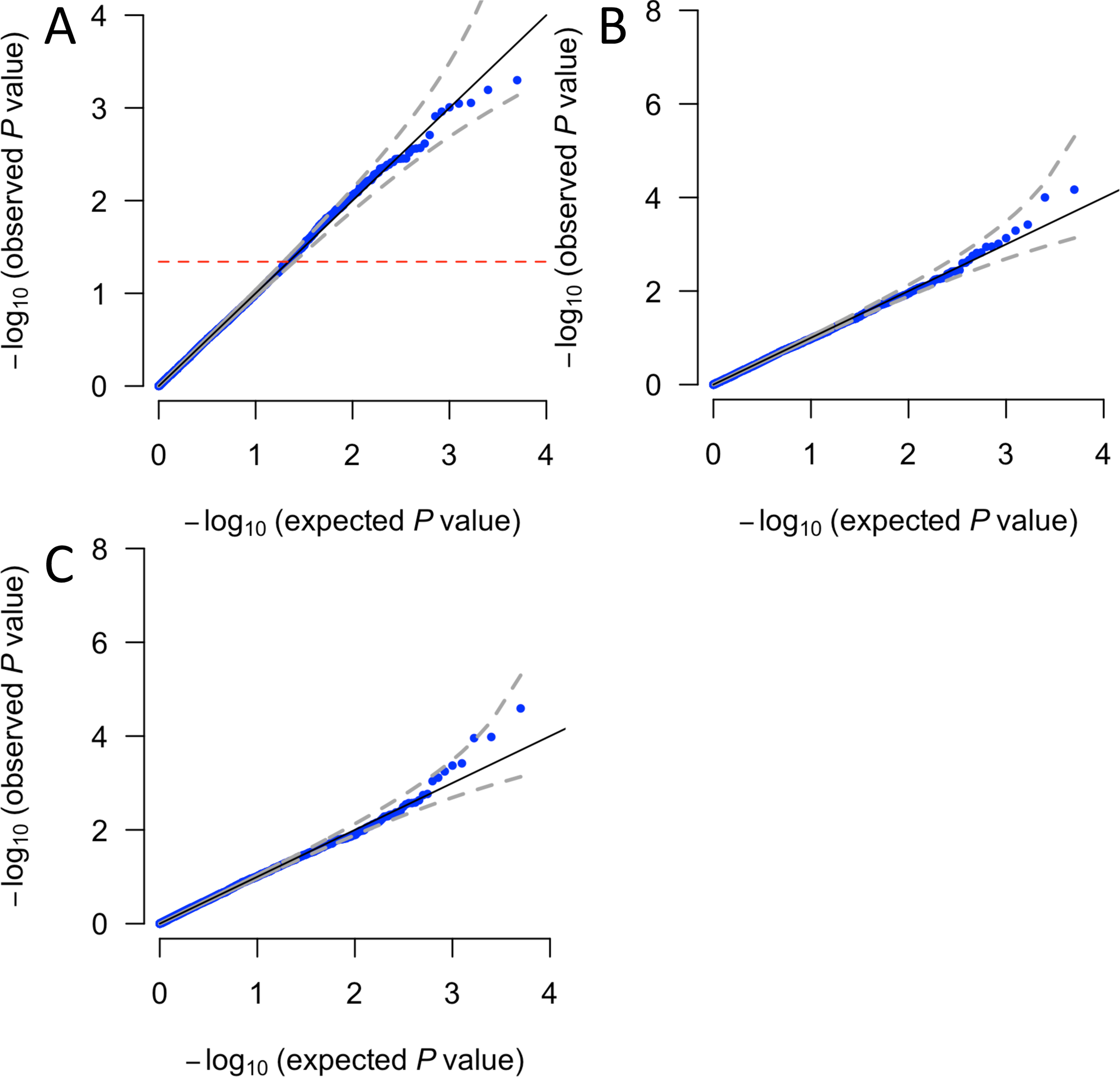

Supplement: S1 Fig — A: Quantile-quantile plot of Spearman correlation test P-values for ranks of Pm and Pv. Quantile-quantile plot of Spearman correlation test P-values for ranks of Pm and Pv. The figure illustrates 5,000 Spearman correlation P values testing for correlation between Pm and and Pv values drawn from a simulated dataset of 44,000 individuals and 50,335 SNPs. In the figure, distribution under the null hypothesis is represented as a black line while its 95% confidence interval is represented as dashed gray lines. The dashed red line represents the correlation P value obtained from the “real data” analysis presented in the main text. B. Quantile-quantile plot of Spearman correlation test P-values for ranks of Pm and Pint. Quantile-quantile plot of Spearman correlation test P-values for ranks of Pm and Pint. The figure illustrates 5,000 Spearman correlation P values testing for correlation between Pm and and Pint values drawn from a simulated dataset of 44,000 individuals and 50,335 SNPs. In the figure, distribution under the null hypothesis is represented as a black line while its 95% confidence interval is represented as dashed gray lines. C. Quantile-quantile plot of Spearman correlation test P-values for ranks of Pint and Pv. Quantile-quantile plot of Spearman correlation test P-values for ranks of Pint and Pv. The figure illustrates 5,000 Spearman correlation P values testing for correlation between Pint and and Pv values drawn from a simulated dataset of 44,000 individuals and 50,335 SNPs. In the figure, distribution under the null hypothesis is represented as a black line while its 95% confidence interval is represented as dashed gray lines. (TIF) [file pgen.1006812.s001.tif]

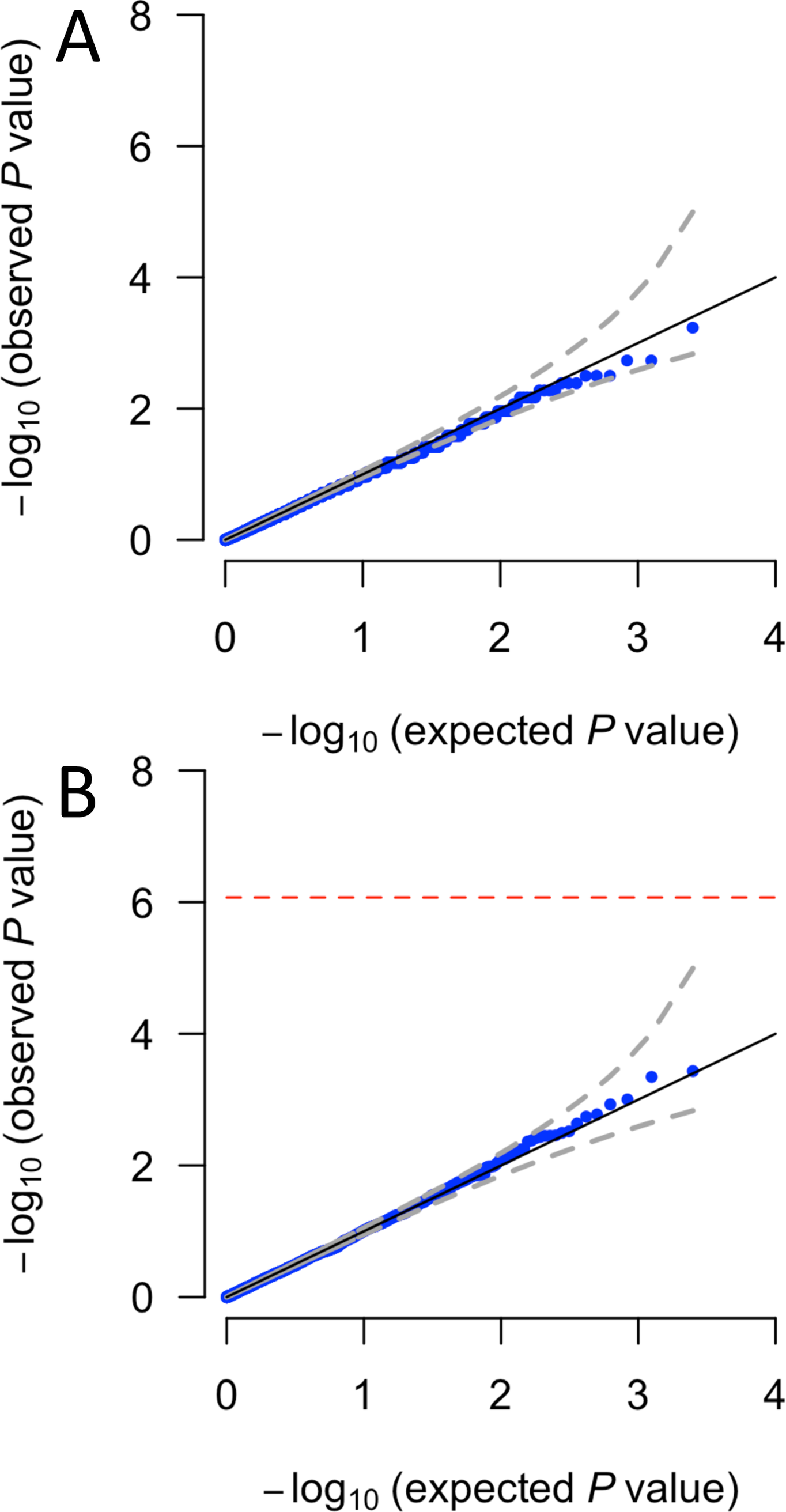

Supplement: S2 Fig — A. Quantile-quantile plot of binomial test P-values for enrichment of variants with Pv<0.05 among variants with Pm<0.05. Quantile-quantile plot of binomial test P-values for enrichment of variants with Pv<0.05 among variants with Pm<0.05. The figure illustrates 2,500 binomial P values testing for enrichment of variants with Pv<0.05 among all variants with Pm<0.05. Pv and and Pm values drawn from a simulated dataset of 44,000 individuals and 50,335 SNPs. In the figure, distribution under the null hypothesis is represented as a black line while its 95% confidence interval is represented as dashed gray lines. B. Quantile-quantile plot of binomial test P-values for enrichment of variants with Pv<0.05 among variants with Pint<0.05. Quantile-quantile plot of binomial test P-values for enrichment of variants with Pv<0.05 among variants with Pint<0.05. The figure illustrates 2,500 binomial P values testing for enrichment of variants with Pv<0.05 among all variants with Pint<0.05. Pv and and Pint values drawn from a simulated dataset of 44,000 individuals and 50,335 SNPs. In the figure, the distribution under the null hypothesis is represented as a black line while its 95% confidence interval is represented as dashed gray lines. The dashed red line represents the correlation P value obtained from the “real data” analysis presented in the main text. (TIF) [file pgen.1006812.s002.tif]

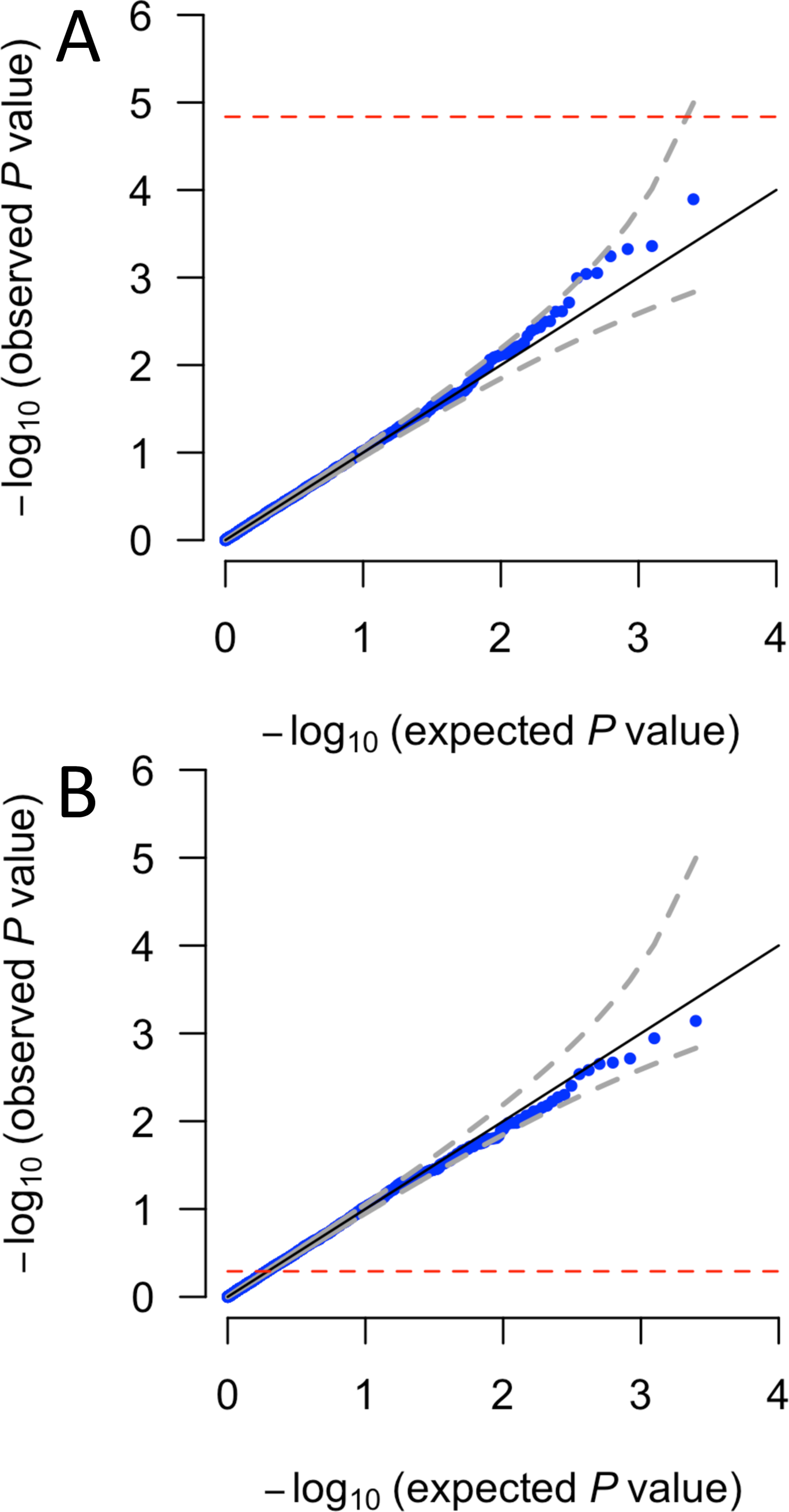

Supplement: S3 Fig — A. Quantile-quantile plot of Mann-Whitney U test P-values for systematic differences in Pv ranks among variants with top ranking and lower ranking Pm values. Quantile-quantile plot of Mann-Whitney U test P-values for systematic differences in Pv ranks among variants with top ranking and lower ranking Pm values. The figure illustrates 2,500 Mann-Whitney U P values testing for systematic differences in Pv ranks among those variants with the most significant Pm values (100th percentile of Pm distribution) and the remaining variants (1–99 percentile of Pm distribution). Pv and and Pm values drawn from a simulated dataset of 44,000 individuals and 50,335 SNPs. In the figure, distribution under the null hypothesis is represented as a black line while its 95% confidence interval is represented as dashed gray lines. The dashed red line represents the correlation P value obtained from the “real data” analysis presented in the main text. B. Quantile-quantile plot of Mann-Whitney U test P-values for systematic differences in Pm ranks among variants with top ranking and lower ranking Pv values. Quantile-quantile plot of Mann-Whitney U test P-values for systematic differences in Pm ranks among variants with top ranking and lower ranking Pv values. The figure illustrates 2,500 Mann-Whitney U P values testing for systematic differences in Pm ranks among those variants with the most significant Pv values (100th percentile of Pv distribution) and the remaining variants (1–99 percentile of Pv distribution). Pv and and Pm values drawn from a simulated dataset of 44,000 individuals and 50,335 SNPs. In the figure, distribution under the null hypothesis is represented as a black line while its 95% confidence interval is represented as dashed gray lines. The dashed red line represents the correlation P value obtained from the “real data” analysis presented in the main text. (TIF) [file pgen.1006812.s003.tif]

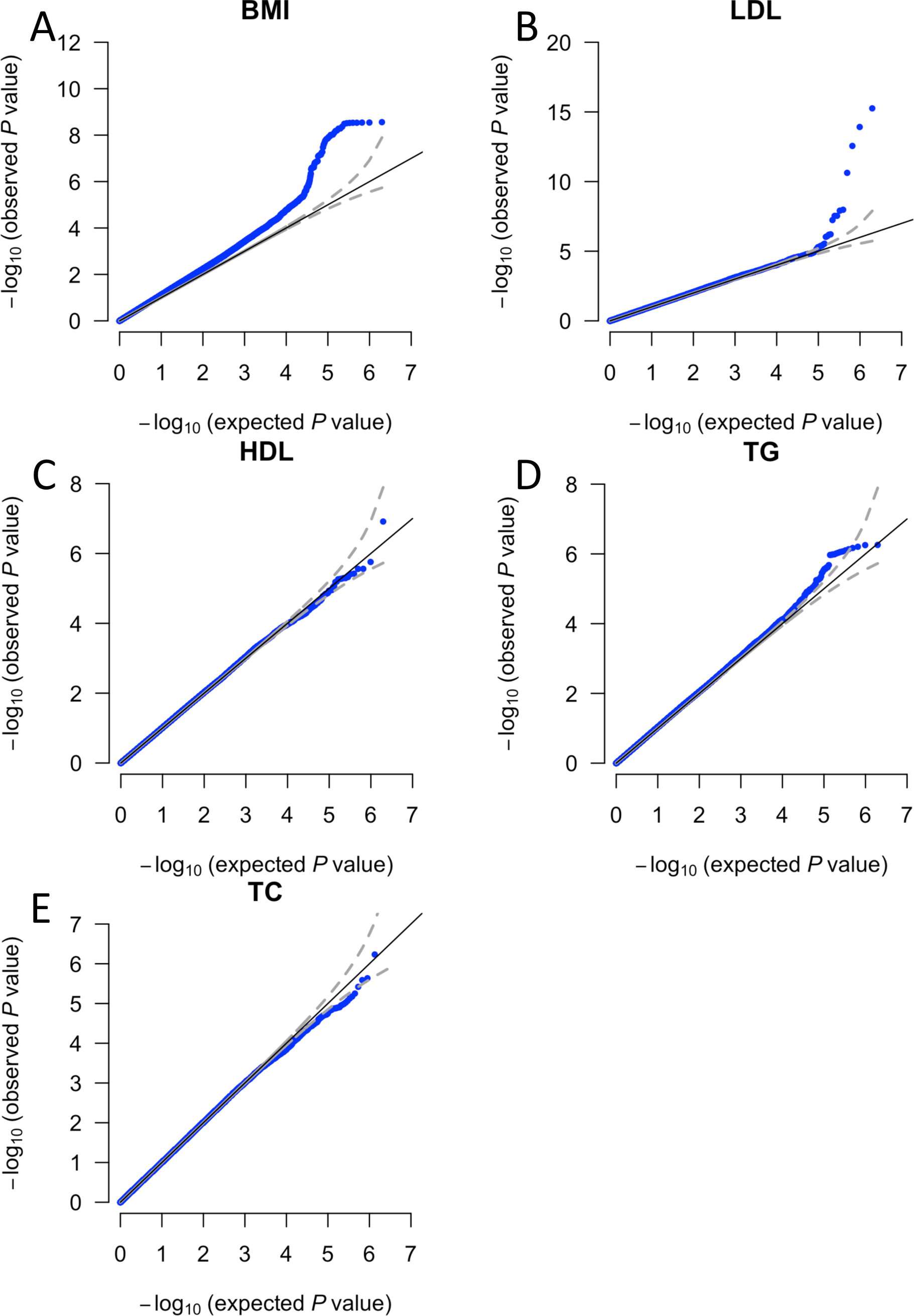

Supplement: S4 Fig — Associations between SNPs and BMI (A), LDL (B), HDL (C), TG (D), TC (E) are presented. Only SNPs with N ≥ 26,000 samples for BMI and N ≥ 24,000 for lipid traits are shown. In each sub-figure, distribution under the null hypothesis is represented as a black line while its 95% confidence interval is represented as dashed gray lines. (TIF) [file pgen.1006812.s004.tif]
