# Supplementary material for: Ranking and characterization of established BMI and lipid associated loci as candidates for gene-environment interactions
Source: PLoS Genet. 2017 Jun 14;13(6):e1006812. doi: 10.1371/journal.pgen.1006812 (PMC5489225; doi:10.1371/journal.pgen.1006812)
Supplement: S1 Text — (PDF) [file pgen.1006812.s008.pdf]

GIANT Consortium contributors

Mette Aadahl[1, 2], Gonçalo R Abecasis[3], Linda S Adair[4], Saima Afaq[5, 6], Uzma Afzal[5, 6], Tarunveer S Ahluwalia[7, 8], Shafqat Ahmad[9], Tamuno Alfred[10, 11], Najaf Amin[12], Juha Auvinen[13, 14], Stephan JL Bakker[15], Damiano Baldassarre[16, 17], Beverley Balkau[18], Stefania Bandinelli[19], Llilda Barata[20], Inês Barroso[21-23], Traci M Bartz[24, 25], Claire Bellis[26, 27], John Beilby[28-30], Richard N Bergman[31], Sven Bergmann[32, 33], Alain G Bertoni[34, 35], Lawrence F Bielak[36], Stephanie Bien[37], Reiner Biffar[38], John Blangero[39], Michael Boehnke[3], Eric Boerwinkle[40], Carsten A Böger[41], Amélie Bonnefond[42], Lori L Bonnycastle[43], Dorret I Boomsma[44, 45], Ingrid B Borecki[20], Judith B Borja[46, 47], Erwin Bottinger[10, 48], Claude Bouchard[49], Søren Brage[50], Marcel Bruinenberg[51], Daniele Braga[52], Jennifer Bragg[53, 54], Brendan M Buckley[55], Fabio Busonero[56], Steve Buyske[57, 58], Gemma Cadby[59], Harry Campbell[60], John C Chambers[61-63], Daniel I Chasman[64-66], Yii-Der Ida Chen[67], Yu-Ching Cheng[68, 69], Peter S Chines[43], Martina Chittani[52], Audrey Y Chu[64, 70], Scott Coggeshall[24], Francis S Collins[43], Richard S Cooper[71], Marilyn Cornelis[72-74], Tanguy Corre[32, 33], Francesco Cucca[56, 75], L Adrienne Cupples[70, 76] , Joanne E Curran[77], Jacek Czajkowski[78], Gert J de Borst[79], Anton JM de Craen[80], Daniele Cusi[81], George Davey Smith[82], George Dedoussis[83], Graciela E Delgado[84], Panagiotis Deloukas[85-87], Ulf de Faire[88], Eco JC de Geus[44, 89], Ayse Demirkan[12, 90], Xuan Deng[91], Marcel den Hoed[50, 92], Hester M den Ruijter[93], Nese Direk[94, 95], Marcus Dörr[96, 97], Nicole Dueker[98], Tapani Ebeling[99, 100], John D Eicher[101], Gudny Eiriksdottir[102], Anna L. Eriksson[103], Joel Eriksson[104], Tõnu Esko[66, 105-107], Jessica D Faul[108], Kristine Færch[8], Mary F Feitosa[20], Luigi Ferrucci[109], Krista Fischer[110], Virginia A Fisher[91], Ian Ford[111], Myriam Fornage[40, 112], Terrence Forrester[113], Caroline S Fox[101], Paul W Franks[9, 73, 114], Timothy M Frayling[115], Philippe Froguel[42, 116], Mao Fu[68], Karl Gertow[117, 118], Christian Gieger[119-121], Bruna Gigante[88], Sven Gläser[96], Nicola Glorioso[122], Jian Gong[37], Penny Gordon-Larsen[4, 123], Mathias Gorski[41, 124], Hans-Jörgen Grabe[125, 126], Mariaelisa Graff[127], Harald Grallert[119, 121, 128], Tanja B Grammer[84], Niels Grarup[7], Paula J Griffin[76], Gerard van Grootheest[129], Vilmundur Gudnason[102, 130], Ulf Gyllensten[131], David Hadley[132], Christopher A Haiman[133], Saskia Haitjema[93], Toomas Haller[105], Göran Hallmans[134], Anders Hamsten[117, 118], Torben Hansen[7], Kennet Harald[135], Marie Neergaard Harder[7], Tamara B Harris[136], Catharina A Hartman[137], Maija Hassinen[138], Nicholas D Hastie[139], Aki S Havulinna[135], James E Hayes[140, 141], Caroline Hayward[139], Nancy L Heard-Costa[70, 142], Andrew C Heath[143], Iris M Heid[144], Dena Hernandez[145], Lucia Hindorff[146], Joel N Hirschhorn[66, 107, 147], Lynne J Hocking[148, 149], Albert Hofman[150, 151], Mette Hollensted[7], Oddgeir L Holmens[152], Christina Holzapfel[119, 153], Georg Homuth[154], Momoko Horikoshi[155, 156], Jouke Jan Hottenga[44, 45], Jie Huang[157], Tao Huang[73], Jennifer E. Huffman[70, 139], Jennie Hui[28, 29, 158], Joseph Hung[159, 160], David J Hunter[161-164], Charlotte Huppertz[44, 89, 165], Cornelia Huth[121, 128], Nina Hutri-Kähönen[166, 167], Kristian Hveem[168], Erik Ingelsson[169-171], Anne U Jackson[3], Alan L James[28, 172, 173], John-Olov Jansson[174], Marjo-Riitta Järvelin[13, 14, 62, 175, 176], Min A Jhun[36], Åsa Johansson[131], Andrew D Johnson [70, 101], Marit E Jørgensen[177], J Wouter Jukema[178-180], Markus Juonala[181, 182], Johanne Marie Justesen[7], Anne E Justice[127], Mika Kähönen[183, 184], Stavroula Kanoni[85], Sharon LR Kardia[36], Magnus Karlsson[185], Tuomas O. Kilpeläinen[7, 50, 186], Leena Kinnunen[187], Mika Kivimäki[188], Marcus E Kleber[84, 189], Robert J Klein[141], Heikki A Koistinen[187, 190, 191], Ivana Kolcic[192], Genovefa Kolovou[193], Pirjo Komulainen[138], Jaspal S Kooner[61, 63, 194], Charles Kooperberg[37], Bernhard K Krämer[195], Kati Kristiansson[135, 196], Diana Kuh[197], Meena Kumari[198], Zoltan Kutalik[33, 199], Johanna Kuusisto[200], Kirsti Kvaløy[168], Markku Laakso[200], Timo A Lakka[138, 201], Claudia Langenberg[50], Lenore J Launer[202], Karin Leander[88], Nanette R Lee[203, 204], Benjamin Lehne[62], Terho Lehtimäki[205, 206], Loic Lemarchand[207], Elise Lim[76], Unhee Lim[207], Lars Lind[208], Cecilia M Lindgren[209-211], Allan Linneberg[1, 212, 213], Ching-Ti Liu[76], Yongmei Liu[34],

Stephane Lobbens[214], Marie Loh[5, 215], Kurt Lohman[216], Ruth JF Loos[50, 186, 217, 218], Mattias Lorentzon[104, 219], Jian'an Luan[50], Robert Luben[220], Gitta Lubke[221], Anja Ludolph-Donislawski[222, 223], Sara Lupoli[52], Leo-Pekka Lyytikäinen[205, 206], Pamela AF Madden[143], Reedik Mägi[105], Anubha Mahajan[209], Massimo Mangino[224, 225], Ani Manichaikul[226], Reija Männikkö[138], Satu Männistö[135], Loic Le Marchand[227], Jonathan Marchini[228], Pedro M Marques-Vidal[229], Michel Marre[230, 231], Jonathan Marten[139], Nicholas G Martin[232], Winfried März[84, 233, 234], Mark I McCarthy[209, 235, 236], Colin A McKenzie[113], Barbara McKnight[24, 25, 237], Dan Mellström[103], Cristina Menni[238], Andres Metspalu[105], Rita PS Middelberg[239], Yuri Milaneschi[129], Karen L Mohlke[240], Keri L Monda[127, 241], Grant W Montgomery[232], Marleen HM De Moor[89, 242], Andrew P Morris[155, 243], Antonella Mulas[56, 75], Martina Müller-Nurasyid[120, 244, 245], AW Musk[28, 158, 246], Narisu Narisu[43], Matthias Nauck[97, 247], Pau Navarro[139], Jennifer A Nettleton[40], Julius S Ngwa[248], Ilja M Nolte[249], Kari E North[250], Jeffrey R O'Connell[68], Claes Ohlsson[104], Albertine J Oldehinkel[251], Matthias Olden[144], Ken K Ong[252], Sandosh Padmanabhan[149, 253], Lyle J Palmer[254], Dorota Pasko[115], Gerard Pasterkamp[93, 255], Lavinia Paternoster[82], Oluf Pedersen[7], Brenda WJH Penninx[129], Jeremiah Perez[76], Markus Perola[135, 196, 256], Louis Pérusse[257, 258], Natalia Pervjakova[110, 259], Annette Peters[121, 128, 245], Ulrike Peters[37], Patricia A Peyser[36], Wouter J Peyrot[129], Charlotta Pisinger[260, 261], Ozren Polasek[60, 192], David J Porteous[149, 262], Inga Prokopenko[263], Bruce M Psaty[264-266], Hannu Puolijoki[267], Lu Qi[73], Qibin Qi[268], Lydia Quaye[224], Olli T Raitakari[269, 270], Tuomo Rankinen[49], DC Rao[143, 271, 272], Laura J Rasmussen-Torvik[72], Rainer Rauramaa[138, 273], Rajesh Rawal[119-121], Frida Renström[9, 134], Treva Rice[143, 271], Paul M Ridker[64, 65], Fernando Rivadeneira[150, 151, 274], Natalia V Rivera[275], Lynda M Rose[64], Igor Rudan[60], Veikko Salomaa[135], Serena Sanna[56], Cinzia Sarti[276], Mark A Sarzynski[49], Naveed Sattar[277], Kai Savonen[138], David Schlessinger[278], Salome Scholtens[279], Claudia Schurmann[10, 11], Robert A Scott[50], William R Scott[62], Bengt Sennblad[117, 118, 280], Alan R Shuldiner[68, 69], Steve Sidney[281], Marten A Siemelink[93], Günther Silbernagel[282], P Eline Slagboom[283], Albert Vernon Smith[102, 130], Blair H Smith[149, 284], Jennifer A Smith[36], Harold Snieder[249], Søren Snitker[68], Thorkild IA Sørensen[2, 7, 82, 285], Tim D Spector[238], Jan A Staessen[286, 287], Alena Stančáková[200], Barbara Sternfeld[281], David J Stott[288], David P Strachan[289], Konstantin Strauch[120, 290], Rona J Strawbridge[117, 118], Heather M Stringham[3], Yun Ju Sung[271], Morris A Swertz[291], Amy J Swift[43], Tuija Tammelin[292], Sian-Tsung Tan[194], Toshiko Tanaka[293], Kent D Taylor[294, 295], Bamidele O Tayo[71], Alexander Teumer[97, 296], Barbara Thorand[121, 128], Dorothée Thuillier[42], Henning Tiemeier[297], Anke Tönjes[298], Elena Tremoli[16, 17], Stella Trompet[80, 178], Jaakko Tuomilehto[187, 299, 300], Jessica Tyrrell[301, 302], André G Uitterlinden[150, 274], Liesbeth Vandenput[104], Pim van der Harst[291, 303, 304], Sander W van der Laan[93], Peter J van der Most[249], Cornelia M van Duijn[12, 151, 305], Jana V van Vliet-Ostaptchouk[306], Sailaja L Vedantam[164, 307], Niek Verweij[303], Henrik Vestergaard[7, 8], Jacqueline M Vink[308, 309], Veronique Vitart[139], Marie-Claude Vohl[258, 310], Uwe Völker[97, 311], Peter Vollenweider[229], Henry Völzke[97, 296], Judith M Vonk[279], Gérard Waeber[229], Melanie Waldenberger[312, 313], Mark Walker[314], Nicholas J Wareham[50], David R Weir[108], RGJ Westendorp, John B Whitfield[232], Sarah Wild[315], Gonneke Willemsen[44, 89], James F Wilson[60, 139], Thomas W Winkler[144], Bruce HR Wolffenbuttel[316], Andrew Wong[197], Tsegaselassie Workalemahu[73], Alan F Wright[139], Joseph Wu[91], Ying Wu[240], Luting Xue[70, 76], Loic Yengo[42], Kristin L Young[123, 127], Weihua Zhang[61, 62], Jing Hua Zhao[50], Wei Zhao[36], M Carola Zillikens[274], Martina E Zimmermann[124], Niha Zubair[37]

Affiliations

1. Research Centre for Prevention and Health, Glostrup University Hospital, Glostrup, 2600, Denmark.
2. Department of Public Health, Faculty of Health and Medical Sciences, University of Copenhagen, Copenhagen, 1014, Denmark
3. Center for Statistical Genetics, Department of Biostatistics, University of Michigan, Ann Arbor, MI, 48109, USA.
4. Department of Nutrition, Gillings School of Global Public Health, University of North Carolina at Chapel Hill, Chapel Hill, NC, 27599, USA.
5. Dept Epidemiology and Biostatistics, School of Public Health, Imperical College London, UK.
6. Cardiology, Ealing Hospital NHS Trust, Middlesex, UK.
7. Novo Nordisk Foundation Center for Basic Metabolic Research, Section of Metabolic Genetics, Faculty of Health and Medical Sciences, University of Copenhagen, Copenhagen, 2100, Denmark.
8. Steno Diabetes Center, Gentofte, DK-2820, Denmark.
9. Department of Clinical Sciences, Genetic and Molecular Epidemiology Unit, Lund University, Malmö, 20502, Sweden.
10. The Charles Bronfman Institute for Personalized Medicine, Icahn School of Medicine at Mount Sinai, New York, NY, USA.
11. The Genetics of Obesity and Related Metabolic Traits Program, Icahn School of Medicine at Mount Sinai, New York, NY, USA.
12. Genetic Epidemiology Unit, Department of Epidemiology, Erasmus MC, Rotterdam, 3015 GE, The Netherlands.
13. Center for Life Course Health Research, Faculty of Medicine, University of Oulu, Oulu, 90014, Finland.
14. Unit of Primary Care, Oulu University Hospital, Oulu, 90220, Finland.
15. Department of Medicine, University Medical Center Groningen, University of Groningen, Groningen, the Netherlands.
16. Dipartimento di Scienze Farmacologiche e Biomolecolari, Università di Milano, Milan , Italy.
17. Centro Cardiologico Monzino, IRCCS, Milan, Italy.
18. INSERM U-1018, CESP, Renal and Cardiovascular Epidemiology, UVSQ-UPS, Villejuif, 94800, France.
19. Geriatric Unit, Azienda Sanitaria Firenze, Florence, 50122, Italy.
20. Department of Genetics, Washington University School of Medicine, St. Louis, MO, 63110, USA.
21. Wellcome Trust Sanger Institute, Hinxton, CB10 1SA, UK.
22. NIHR Cambridge Biomedical Research Centre, Institute of Metabolic Science, Addenbrooke’s Hospital, Cambridge, CB2 0QQ, UK.
23. The University of Cambridge Metabolic Research Laboratories, Wellcome Trust-MRC Institute of Metabolic Science, Cambridge, CB2 0QQ, UK.
24. Department of Biostatistics, University of Washington, Seattle, WA 98195.
25. Cardiovascular Health Research Unit, Department of Medicine, University of Washington, Seattle, WA 98101.
26. Human Genetics, Genome Institute of Singapore, Agency for Science, Technology and Research of Singapore, Singapore.
27. Genomics Research Centre, Institute of Health and Biomedical Innovation, Queensland University of Technology, Brisbane, Queensland 4001, Australia.
28. Busselton Population Medical Research Institute, Nedlands, WA 6009, Australia.
29. PathWest Laboratory Medicine of WA, Sir Charles Gairdner Hospital, Nedlands, WA 6009, Australia.
30. School of Pathology and Laboratory Medicine, The University of Western Australia, Crawley, WA 6009, Australia.

31. Diabetes and Obesity Research Institute, Cedars-Sinai Medical Center, Los Angeles, CA, 90048, USA.
32. Department of Medical Genetics, University of Lausanne, Lausanne, 1015, Switzerland.
33. Swiss Institute of Bioinformatics, Lausanne, 1015, Switzerland.
34. Department of Epidemiology and Prevention, Division of Public Health Sciences, Wake Forest School of Medicine, Winston-Salem, NC, 27157, USA.
35. Department of Internal Medicine, Wake Forest School of Medicine, Winston-Salem, NC, 27157, USA.
36. Department of Epidemiology, School of Public Health, University of Michigan, Ann Arbor, MI, 48109, USA.
37. Division of Public Health Sciences, Fred Hutchinson Cancer Research Center, Seattle, WA, 98109-1024, USA.
38. Clinic for Prosthetic Dentistry, Gerostomatology and Material Science, University Medicine Greifswald, Germany.
39. Texas Biomedical Research Institute, San Antonio, TX, 78245, USA.
40. Division of Epidemiology, Human Genetics, and Environmental Sciences, University of Texas Health Science Center at Houston, Houston, TX, 77030, USA.
41. Department of Nephrology, University Hospital Regensburg, Regensburg, Germany.
42. University of Lille, CNRS, Institut Pasteur de Lille, UMR 8199 - EGID, Lille, 59019, France.
43. Medical Genomics and Metabolic Genetics Branch, National Human Genome Research Institute, NIH, Bethesda, MD, 20892, USA.
44. Department of Biological Psychology, Vrije Universiteit, Amsterdam, 1081 BT, The Netherlands.
45. NCA Institute, VU University & VU Medical Center, Amsterdam, 1081 HV, The Netherlands.
46. USC-Office of Population Studies Foundation, Inc., University of San Carlos, Cebu City, 6000, Philippines.
47. Department of Nutrition and Dietetics, University of San Carlos, Cebu City, 6000, Philippines.
48. Department of Pharmacology and Systems Therapeutics, Icahn School of Medicine at Mount Sinai, New York, NY, USA.
49. Human Genomics Laboratory, Pennington Biomedical Research Center, Baton Rouge, LA, 70808, USA.
50. MRC Epidemiology Unit, Institute of Metabolic Science, University of Cambridge, Cambridge, CB2 0QQ, UK.
51. Lifelines Cohort Study, PO Box 30001, 9700 RB Groningen, the Netherlands.
52. Dept. Health Sciences, University of Milan, Via A. Di Rudiní, 8 20142, Milano, Italy.
53. Internal Medicine - Nephrology, University of Michigan, Ann Arbor, Michigan, USA.
54. Department of Biostatistics and Center for Statistical Genetics, University of Michigan, Ann Arbor, MI 48109, USA.
55. Department of Pharmacology and Therapeutics, University College Cork, Ireland.
56. Istituto di Ricerca Genetica e Biomedica (IRGB), Consiglio Nazionale Delle Ricerche (CNR), Cittadella Universitaria di Monserrato, SS554 Km 4500, 09042, Monserrato, Italy.
57. Department of Genetics, Rutgers University, Piscataway, NJ, 08854, USA.
58. Department of Statistics and Biostatistics, Rutgers University, Piscataway, NJ, 08854, USA.
59. Centre for Genetic Origins of Health and Disease, University of Western Australia, Crawley, WA 6009, Australia.
60. Centre for Global Health Research, Usher Institute for Population Health Sciences and Informatics, Teviot Place, Edinburgh, EH8 9AG, Scotland.
61. Department of Cardiology, Ealing Hospital HNS Trust, Middlesex, UB1 3HW, United Kingdom.

62. Department of Epidemiology and Biostatistics, School of Public Health, Imperial College London, London W2 1PG, UK.
63. Imperial College Healthcare NHS Trust, London W12 0HS, UK.
64. Division of Preventive Medicine, Brigham and Women's Hospital, Boston, MA, 02215, USA.
65. Harvard Medical School, Boston, MA, 02115, USA.
66. Broad Institute of the Massachusetts Institute of Technology and Harvard University, Cambridge, MA, 2142, USA.
67. Institute for Translational Genomics and Population Sciences, Los Angeles BioMedical Research Institute and Department of Pediatrics, Harbor-UCLA, Torrance, CA 90502, USA.
68. Division of Endocrinology, Diabetes, and Nutrition, University of Maryland School of Medicine, Baltimore, MD, 21201, USA.
69. Veterans Affairs Maryland Health Care System, University of Maryland, Baltimore, MD, 21201, USA.
70. National Heart, Lung, and Blood Institute, Framingham Heart Study, Framingham, MA, 01702, USA.
71. Department of Public Health Sciences, Stritch School of Medicine, Loyola University of Chicago, Maywood, IL 61053 USA.
72. Department of Preventive Medicine, Northwestern University Feinberg School of Medicine, Chicago, IL, 60611, USA.
73. Department of Nutrition, Harvard T.H. Chan School of Public Health, Boston, MA, 02115, USA.
74. Channing Division of Network Medicine, Department of Medicine, Brigham and Women's Hospital and Harvard Medical School, Boston, MA, 02115, USA.
75. Dipartimento di Scienze Biomediche, Università degli Studi di Sassari, Sassari, 07100, Italy.
76. Department of Biostatistics, Boston University School of Public Health, Boston, MA, 02118, USA.
77. South Texas Diabetes and Obesity Institute, University of Texas Rio Grande Valley, Brownsville, TX.
78. Division of Statistical Genomics, Department of Genetics, Washington University School of Medicine; St. Louis, MO, 63108 USA.
79. Department of Vascular Surgery, Division of Surgical Specialties, UMC Utrecht, the Netherlands.
80. Department of Gerontology and Geriatrics, Leiden University Medical Center, The Netherlands.
81. Sanipedia srl, Bresso (Milano), Italy and Institute of Biomedical Technologies National Centre of Research Segrate (Milano), Italy.
82. MRC Integrative Epidemiology Unit & School of Social and Community Medicine, University of Bristol, Bristol, BS82BN, UK.
83. Department of Nutrition and Dietetics, School of Health Science and Education, Harokopio University, Athens, Greece.
84. Vth Department of Medicine, Medical Faculty Mannheim, Heidelberg University, Mannheim, 68167, Germany.
85. William Harvey Research Institute, Barts and The London School of Medicine and Dentistry, Queen Mary University of London, London, UK.
86. Wellcome Trust Sanger Institute, Hinxton, Cambridge, UK.
87. Princess Al-Jawhara Al-Brahim Centre of Excellence in Research of Hereditary Disorders (PACER-HD), King Abdulaziz University, Jeddah, Saudi Arabia.
88. Unit of Cardiovascular Epidemiology, Institute of Environmental Medicine, Karolinska Institutet, Stockholm, Sweden.
89. EMGO+ Institute, Vrije Universiteit & VU University Medical Center, Amsterdam, 1081 BT, The Netherlands.

90. Department of Human Genetics, Leiden University Medical Center, Leiden, 2333, The Netherlands.
91. Department of Biostatistics, Boston University School of Public Health, Boston, MA 02118.
92. Department of Medical Sciences, Molecular Epidemiology and Science for Life Laboratory, Uppsala University, Uppsala, 75141, Sweden.
93. Laboratory of Experimental Cardiology, Department of Cardiology, Division Heart & Lungs, UMC Utrecht, the Netherlands.
94. Department of Epidemiology, Erasmus Medical Center, Rotterdam, Netherlands.
95. Department of Psychiatry, Dokuz Eylul University, Izmir, Turkey.
96. Department of Internal Medicine B, University Medicine Greifswald, Greifswald, 17475, Germany.
97. DZHK (German Center for Cardiovascular Research), partner site Greifswald, Greifswald, 17489, Germany
98. University of Maryland School of Medicine, Department of Epidemiology & Public Health, Baltimore, MD, 21201, USA.
99. Department of Medicine, Oulu University Hospital, Oulu, 90220, Finland.
100. Institute of Clinical Medicine, Faculty of Medicine, University of Oulu, Oulu, 90014, Finland.
101. Population Sciences Branch, National Heart, Lung, and Blood Institute, National Institutes of Health, The Framingham Heart Study, Framingham, MA, 01702, USA.
102. Icelandic Heart Association, Kopavogur, 201, Iceland.
103. Centre for Bone and Arthritis Research, Department of Internal Medicine and Clinical Nutrition, Institute of Medicine, Sahlgrenska Academy at the University of Gothenburg, Gothenburg, Sweden.
104. Centre for Bone and Arthritis Research, Department of Internal Medicine and Clinical Nutrition, Institute of Medicine, Sahlgrenska Academy, University of Gothenburg, Gothenburg, 413 45, Sweden.
105. Estonian Genome Center, University of Tartu, Tartu, 51010, Estonia.
106. Division of Endocrinology, Boston Children's Hospital, Boston, MA, 02115, USA.
107. Department of Genetics, Harvard Medical School, Boston, MA, 02115, USA.
108. Survey Research Center, Institute for Social Research, University of Michigan, Ann Arbor, MI, 48104, USA.
109. Translational Gerontology Branch, National Institute on Aging, Baltimore MD, USA.
110. Estonian Genome Center, University of Tartu, Tartu 51010, Estonia.
111. Robertson Centre for Biostatistics, University of Glasgow, United Kingdom.
112. Institute of Molecular Medicine, University of Texas Health Science Center at Houston, Houston, TX, 77030, USA.
113. Tropical Metabolism Research Unit, Tropical Medicine Research Institute, University of the West Indies, Mona, JMAAW15 Jamaica.
114. Department of Public Health & Clinical Medicine, Umeå University, Umeå, 90187, Sweden.
115. Genetics of Complex Traits, University of Exeter Medical School, University of Exeter, Exeter, EX2 5DW, UK.
116. Hammersmith Hospital, London, W12 0HS, United Kingdom.
117. Cardiovascular Medicine Unit, Department of Medicine Solna, Karolinska Institutet, Stockholm, Sweden.
118. Center for Molecular Medicine, Karolinska University Hospital Solna, Stockholm, Sweden.
119. Research Unit of Molecular Epidemiology, Helmholtz Zentrum München - German Research Center for Environmental Health, Neuherberg, 85764, Germany.
120. Institute of Genetic Epidemiology, Helmholtz Zentrum München, German Research Center for Environmental Health, Neuherberg, 85764, Germany.
121. Institute of Epidemiology II, Helmholtz Zentrum München-German Research Center for Environmental Health, Neuherberg, 85764, Germany.

122. Hypertension and Related Disease Centre, AOU-University of Sassari.
123. Carolina Population Center, University of North Carolina at Chapel Hill, Chapel Hill, NC, 27514, USA.
124. Department of Genetic Epidemiology, Institute of Epidemiology and Preventive Medicine, University of Regensburg, D-93053 Regensburg, Germany.
125. Department of Psychiatry and Psychotherapy, University Medicine Greifswald, Germany.
126. German Center for Neurodegenerative Diseases (DZNE), Site Rostock/ Greifswald, Germany.
127. Department of Epidemiology, Gillings School of Global Public Health, University of North Carolina at Chapel Hill, Chapel Hill, NC, 27599, USA.
128. German Center for Diabetes Research (DZD), München-Neuherberg, 85764, Germany.
129. Department of Psychiatry, EMGO Institute for Health and Care Research and Neuroscience Campus Amsterdam, VU University Medical Center/GGZ InGeest, Amsterdam, 1081 HL, The Netherlands
130. Faculty of Medicine, University of Iceland, Reykjavik, 101, Iceland.
131. Department of Immunology, Genetics and Pathology, Uppsala University, Uppsala, 751 08, Sweden.
132. Division of Population Health Sciences and Education, St. George's, University of London, London, SW17 0RE, United Kingdom.
133. Department of Preventive Medicine, Norris Comprehensive Cancer Center, Keck School of Medicine, University of Southern California, Los Angeles, CA, 90089, USA.
134. Department of Biobank Research, Umeå University, Umeå, 90187, Sweden.
135. National Institute for Health and Welfare, Department of Health, Helsinki, FI-00271, Finland.
136. Laboratory of Epidemiology and Population Science, National Institute on Aging, Bethesda, MD, 20892, USA.
137. Department of Psychiatry, University of Groningen, University Medical Center Groningen, Groningen, 9700 RB, The Netherlands.
138. Kuopio Research Institute of Exercise Medicine, Kuopio, 70100, Finland .
139. MRC Human Genetics Unit, Institute of Genetics and Molecular Medicine, University of Edinburgh, Western General Hospital, Edinburgh, EH4 2XU, United Kingdom.
140. Cell and Developmental Biology Graduate Program, Weill Cornell Graduate School of Medical Sciences, Cornell University, New York, NY, 10021, USA.
141. Icahn Institute for Genomics and Multiscale Biology, Icahn School of Medicine at Mount Sinai, New York, NY, 10029, USA.
142. Department of Neurology, Boston University School of Medicine, Boston, MA, 02118, USA.
143. Department of Psychiatry, Washington University School of Medicine, St. Louis, MO.
144. Department of Genetic Epidemiology, University of Regensburg, Regensburg, 93053, Germany.
145. Laboratory of Neurogenetics, National Institute on Aging, Bethesda, MD, 20892, USA.
146. Division of Genomic Medicine, National Human Genome Research Institute, National Institutes of Health, Bethesda, MD, 20892, USA.
147. Divisions of Endocrinology and Genetics and Center for Basic and Translational Obesity Research, Boston Children's Hospital, Boston, MA, 02115, USA.
148. Musculoskeletal Research Programme, Division of Applied Medicine, University of Aberdeen, Foresterhill, Aberdeen, AB25 2ZD, United Kingdom.
149. Generation Scotland, Centre for Genomic and Experimental Medicine, University of Edinburgh, Edinburgh, EH4 2XU, United Kingdom.
150. Department of Epidemiology, Erasmus MC, Rotterdam, 3015 GE, The Netherlands.
151. Netherlands Consortium for Healthy Aging, Leiden University Medical Center, Leiden, 2300 RC, The Netherlands.

152. St. Olav Hospital, Trondheim University Hospital, Trondheim, 7030, Norway.
153. Institute for Nutritional Medicine, Klinikum Rechts der Isar, Technische Universität München, Munich, 81675, Germany.
154. Interfaculty Institute for Genetics and Functional Genomics, University Medicine Greifswald, Germany.
155. Wellcome Trust Centre for Human Genetics, University of Oxford, Oxford, OX3 7BN, UK.
156. Oxford Centre for Diabetes, Endocrinology and Metabolism, University of Oxford, Churchill Hospital, Oxford, OX3 7LJ, UK.
157. Department of Human Genetics, Wellcome Trust Sanger Institute, Hinxton, Cambridge, CB10 1SA, United Kingdom.
158. School of Population Health, The University of Western Australia, Crawley, WA 6009, Australia.
159. School of Medicine and Pharmacology, The University of Western Australia, 25 Stirling Hwy, Crawley, WA 6009, Australia.
160. Discovery and refinement of loci associated with lipid levels. Nat Genet, 2013. **45**: p. 1274-83.
161. Department of Epidemiology, Harvard T.H. Chan School of Public Health, Boston, MA 02115 USA.
162. Department of Nutrition, Harvard T.H. Chan School of Public Health, Boston, MA 02115, USA.
163. Channing Division of Network Medicine, Department of Medicine, Brigham and Women's Hospital and Harvard Medical School, Boston, MA 02115 USA.
164. Broad Institute of Harvard and MIT, Cambridge, MA 02142 USA.
165. Department of Public and Occupational Health, VU University Medical Center, Amsterdam, 1081 BT, The Netherlands.
166. Department of Pediatrics, Tampere University Hospital, Tampere, 33521, Finland.
167. Department of Pediatrics, University of Tampere School of Medicine, Tampere, 33014, Finland.
168. HUNT Research Centre, Department of Public Health and General Practice, Norwegian University of Science and Technology, Levanger, 7600, Norway.
169. Department of Medical Sciences, Molecular Epidemiology, Uppsala University, Uppsala, 751 85, Sweden.
170. Department of Medicine, Division of Cardiovascular Medicine, Stanford University School of Medicine, Stanford, CA 94305, USA.
171. Science for Life Laboratory, Uppsala University, Uppsala, 750 85, Sweden.
172. Department of Pulmonary Physiology and Sleep Medicine, Sir Charles Gairdner Hospital, Nedlands, WA 6009, Australia.
173. School of Medicine and Pharmacology, The University of Western Australia, Crawley, WA 6009, Australia.
174. Department of Physiology, Institute of Neuroscience and Physiology, Sahlgrenska Academy, University of Gothenburg, Gothenburg, 41345, Sweden.
175. Biocenter Oulu, University of Oulu, Oulu, 90220, Finland.
176. MRC-PHE Centre for Environment and Health, Imperial College London, London, SW7 2AZ, UK.
177. Steno Diabetes Center, Gentofte, Denmark.
178. Department of Cardiology, Leiden University Medical Center, The Netherlands.
179. Durrer Center for Cardiogenetic Research, Amsterdam, The Netherlands.
180. Interuniversity Cardiology Institute of the Netherlands, Utrecht, The Netherlands.
181. Department of Medicine, University of Turku, Turku, FI-20520, Finland.
182. Division of Medicine, Turku University Hospital, Turku, FI-20521, Finland.
183. Department of Clinical Physiology, Tampere University Hospital, Tampere, FI-33521, Finland.
184. Department of Clinical Physiology, University of Tampere School of Medicine, Tampere, FI-33014, Finland.

185. Clinical and Molecular Osteoporosis Research Unit, Department of Orthopedics and Clinical Sciences, Skåne University Hospital, Lund University, Malmö, Sweden.
186. The Department of Preventive Medicine, The Icahn School of Medicine at Mount Sinai, New York, NY, 10029, USA.
187. National Institute for Health and Welfare, Department of Health, FI-00271, Helsinki, Finland.
188. Department of Epidemiology and Public Health, University College London, London, WC1E 6BT, United Kingdom.
189. Institute of Nutrition, Friedrich Schiller University Jena, Jena, 07743, Germany.
190. Department of Medicine and Abdominal Center: Endocrinology, University of Helsinki and Helsinki University Central Hospital, Helsinki, FI-00029, Finland.
191. Minerva Foundation Institute for Medical Research, Helsinki, FI-00290, Finland.
192. Department of Public Health, Faculty of Medicine, University of Split, Split, 21000, Croatia.
193. Department of Cardiology, Onassis Cardiac Surgery Center, Athens, Greece.
194. National Heart and Lung Institute, Imperial College London, W12 0NN, UK.
195. Vth Department of Medicine, Medical Faculty Mannheim, Heidelberg University, Mannheim, Germany.
196. Institute for Molecular Medicine Finland, University of Helsinki, Helsinki, FI-00290, Finland.
197. MRC Unit for Lifelong Health and Ageing at UCL, London, WC1B 5JU, United Kingdom.
198. ISER, University of Essex, Colchester, Essex, CO43SQ, United Kingdom.
199. Institute of Social and Preventive Medicine, Lausanne University Hospital, Lausanne, 1010, Switzerland.
200. Department of Medicine, University of Eastern Finland and Kuopio University Hospital, Kuopio, 70210, Finland.
201. Institute of Biomedicine, Physiology, University of Eastern Finland, Kuopio Campus, 70210, Finland.
202. Neuroepidemiology Section, National Institute on Aging, National Institutes of Health, Bethesda, MD, 20892-9205, USA.
203. USC-Office of Population Studies Foundation, Inc., University of San Carlos, Cebu City 6000, Philippines.
204. Department of Anthropology, Sociology and History, University of San Carlos, Cebu City 6000, Philippines.
205. Department of Clinical Chemistry, Fimlab Laboratories, Tampere, FI-33101, Finland.
206. Department of Clinical Chemistry, University of Tampere School of Medicine, Tampere, FI-33014, Finland.
207. Epidemiology Program, University of Hawaii Cancer Center, Honolulu, HI, 98613, USA.
208. Department of Medical Sciences, Cardiovascular Epidemiology, Uppsala University, Uppsala 751 85, Sweden.
209. Wellcome Trust Centre for Human Genetics, University of Oxford, Oxford, OX3 7BN, UK.
210. Program in Medical and Population Genetics, Broad Institute, Cambridge, MA, 02142, USA.
211. The Big Data Institute, University of Oxford, Oxford, OX1 2JD, UK.
212. Department of Clinical Experimental Research, Rigshospitalet, Glostrup, 2600, Denmark.
213. Department of Clinical Medicine, Faculty of Health and Medical Sciences, University of Copenhagen, Copenhagen, 2200, Denmark.
214. University of Lille, CNRS, Institut Pasteur of Lille, UMR 8199 - EGID, Lille, France.
215. Translational Laboratory in Genetic Medicine (TLGM), Agency for Science, Technology and Research (A*STAR), 8A Biomedical Grove, Immunos, Level 5, Singapore 138648.

216. Department of Biostatistical Sciences, Division of Public Health Sciences, Wake Forest School of Medicine, Winston-Salem, NC, 27157, USA.
217. Genetics of Obesity and Related Metabolic Traits Program, Charles Bronfman Institute for Personalized Medicine, Icahn School of Medicine at Mount Sinai, New York, NY, 10029, USA.
218. The Mindich Child Health and Development Institute, Icahn School of Medicine at Mount Sinai, New York, NY, 10029, USA.
219. Geriatric Medicine, Sahlgrenska University Hospital, Mölndal, 43180, Sweden.
220. Department of Public Health and Primary Care, University of Cambridge, Cambridge, CB1 8RN, United Kingdom.
221. Department of Psychology, University of Notre Dame, Notre Dame, USA.
222. Institute of Genetic Epidemiology, Helmholtz Zentrum München - German Research Center for Environmental Health, D-85764 Neuherberg, Germany.
223. Institute of Medical Informatics, Biometry and Epidemiology, Chair of Genetic Epidemiology, Ludwig-Maximilians-Universität, D-81377 Munich, Germany.
224. Department of Twin Research and Genetic Epidemiology, King's College London, London, SE1 7EH, UK.
225. National Institute for Health Research Biomedical Research Centre at Guy's and St. Thomas' Foundation Trust, London, SE1 9RT, UK.
226. Center for Public Health Genomics and Biostatistics Section, Department of Public Health Sciences, University of Virginia, Charlottesville, Virginia 22903.
227. Epidemiology Program, University of Hawaii Cancer Center, Honolulu, HI 96813, USA.
228. Department of Statistics, University of Oxford, Oxford, UK.
229. Department of Internal Medicine, Internal Medicine, Lausanne University Hospital, Lausanne, 1011, Switzerland.
230. INSERM U-1138, Équipe 2: Pathophysiology and Therapeutics of Vascular and Renal diseases Related to Diabetes, Centre de Recherche des Cordeliers, Paris, 75006, France.
231. Department of Endocrinology, Diabetology, Nutrition, and Metabolic Diseases, Bichat Claude Bernard Hospital, Paris, 75018, France.
232. Genetic Epidemiology, QIMR Berghofer Medical Research Institute, Brisbane, 4029, Australia.
233. Synlab Academy, Synlab Services LLC, Mannheim, 68161, Germany.
234. Clinical Institute of Medical and Chemical Laboratory Diagnostics, Medical University of Graz, Graz, 8010, Austria.
235. Oxford Centre for Diabetes, Endocrinology and Metabolism, University of Oxford, Churchill Hospital, Oxford, OX3 7LJ, UK.
236. Oxford NIHR Biomedical Research Centre, Oxford, OX3 7LJ, UK.
237. Program in Biostatistics and Biomathematics, Fred Hutchinson Cancer Research Center, Seattle, WA 98109.
238. Department of Twin Research and Genetic Epidemiology, King's College London, London, UK.
239. Genetic Epidemiology, QIMR Berghofer Medical Research Institute, Brisbane, Australia.
240. Department of Genetics, University of North Carolina, Chapel Hill, NC, 27599, USA.
241. Center for Observational Research, Amgen Inc., Thousand Oaks, CA, 91320-1799, USA.
242. Section of Clinical Child and Family Studies, Department of Educational and Family Studies, Vrije Universiteit, Amsterdam, 1081 BT, The Netherlands.
243. Department of Biostatistics, University of Liverpool, Liverpool L69 3GL, UK.
244. Department of Medicine I, Ludwig-Maximilians-Universität, Munich, 81377, Germany.
245. DZHK (German Centre for Cardiovascular Research), partner site Munich Heart Alliance, Munich, 80802, Germany.

246. Department of Respiratory Medicine, Sir Charles Gairdner Hospital, Nedlands, WA 6009, Australia.
247. Institute of Clinical Chemistry and Laboratory Medicine, University Medicine Greifswald, Greifswald, 17475, Germany.
248. Howard University, Department of Internal Medicine, Washington, DC, 20060, USA.
249. Department of Epidemiology, University of Groningen, University Medical Center Groningen, Groningen, 9700 RB, The Netherlands.
250. Carolina Center for Genome Sciences, Gillings School of Global Public Health, University of North Carolina at Chapel Hill, Chapel Hill, North Carolina, 27599, USA.
251. Interdisciplinary Center Psychopathology and Emotion Regulation (ICPE), University of Groningen, University Medical Center Groningen, Groningen, 9700 RB, The Netherlands.
252. MRC Epidemiology Unit, University of Cambridge School of Clinical Medicine, Institute of Metabolic Science, Cambridge, CB2 0QQ, UK.
253. Institute of Cardiovascular and Medical Sciences, BHF Glasgow Cardiovascular Research Centre, University of Glasgow, Glasgow, G12 8QQ, United Kingdom.
254. School of Public Health, University of Adelaide, Adelaide, SA 5005, Australia.
255. Laboratory of Clinical Chemistry and Hematology, Division Laboratories & Pharmacy, UMC Utrecht, the Netherlands.
256. University of Tartu, Estonian Genome Centre, Tartu, 51010, Estonia.
257. Department of Kinesiology, Laval University, Quebec, G1V 0A6, Canada.
258. Institute of Nutrition and Functional Foods, Quebec, G1V 0A6, Canada.
259. Department of Biotechnology, Institute of Molecular and Cell Biology, University of Tartu, Tartu 51010, Estonia.
260. Research Center for Prevention and Health, Glostrup Hospital, Glostrup Denmark.
261. Department of Public Health, Faculty of Health Sciences, University of Copenhagen, Denmark.
262. Centre for Genomic and Experimental Medicine, Institute of Genetics and Molecular Medicine, University of Edinburgh, Edinburgh, EH4 2XU, United Kingdom.
263. Genomics of Common Disease, Imperial College London, London, SW7 2AZ, United Kingdom.
264. Department of Medicine, University of Washington, Seattle, WA 98195.
265. Department of Epidemiology, University of Washington, Seattle, WA 98101.
266. Group Health Research Institute, Group Health Cooperative, Seattle, WA 98101.
267. South Ostrobothnia Central Hospital, Seinäjoki, 60220, Finland.
268. Department of Epidemiology and Population Health, Albert Einstein College of Medicine, Bronx, NY, 10461, USA.
269. Department of Clinical Physiology and Nuclear Medicine, Turku University Hospital, Turku, FI-2051, Finland.
270. Research Centre of Applied and Preventive Cardiovascular Medicine, University of Turku, Turku, FI-20520, Finland.
271. Division of Biostatistics, Washington University School of Medicine, St Louis, MO.
272. Department of Genetics, Washington University School of Medicine, St. Louis, MO.
273. Department of Clinical Physiology and Nuclear Medicine, Kuopio University Hospital, Kuopio, 70210, Finland
274. Department of Internal Medicine, Erasmus MC, Rotterdam, 3015 GE, The Netherlands.
275. Karolinska Institutet, Respiratory Unit, Department of Medicine Solna, Stockholm, 17177, Sweden.
276. Social Services and Health Care Department, City of Helsinki, Helsinki, FI-00099, Finland.
277. BHF Glasgow Cardiovascular Research Centre, Faculty of Medicine, Glasgow, United Kingdom.
278. Laboratory of Genetics, National Institute on Aging, National Institutes of Health, Baltimore, MD, USA.

279. Department of Epidemiology, University of Groningen, University Medical Center Groningen, The Netherlands.
280. Science for Life Laboratory, Karolinska Institutet, Stockholm, Sweden.
281. Division of Resesarch, Kaiser Permanente Northern California, Oakland, CA, 94612, USA.
282. Division of Angiology, Department of Internal Medicine, Medical University Graz, 8010, Austria.
283. Department of Molecular Epidemiology, Leiden University Medical Center, Leiden , The Netherlands.
284. School of Medicine, University of Dundee, Ninewells Hospital and Medical School, Dundee, DD2 4BF, Scotland.
285. Department of Clinical Epidemiology, Bispebjerg and Frederiksberg Hospitals, The Capital Region, Copenhagen, 2000, Denmark.
286. Research Unit Hypertension and Cardiovascular Epidemiology, Department of Cardiovascular Science , University of Leuven, Campus Sint Rafael, Kapucijnenvoer 35, Leuven; Belgium.
287. R&D VitaK Group, Maastricht University, Brains Unlimited Building, Oxfordlaan 55, Maastricht, The Netherlands.
288. Institute of Cardiovascular and Medical Sciences, Faculty of Medicine, University of Glasgow, United Kingdom.
289. Population Health Research Institute, St. George's University of London, London, SW17 0RE, United Kingdom.
290. Institute of Medical Informatics, Biometry and Epidemiology, Chair of Genetic Epidemiology, Ludwig-Maximilians-Universität, Munich, 81377, Germany.
291. Department of Genetics, University of Groningen, University Medical Center Groningen, the Netherlands.
292. LIKES Research Center for Sport and Health Sciences, Jyväskylä, 40720, Finland.
293. Translational Gerontology Branch, National Institute on Aging, Baltimore, MD, 21225, USA.
294. Center for Translational Genomics and Population Sciences, Los Angeles Biomedical Research Institute at Harbor/UCLA Medical Center, Torrance, CA, USA.
295. Department of Pediatrics, University of California Los Angeles, Los Angeles, CA
296. Institute for Community Medicine, University Medicine Greifswald, Greifswald, 17475, Germany.
297. Department of Psychiatry Erasmus Medical Center, Rotterdam, Netherlands.
298. University of Leipzig, Medical Department, Leipzig, 04103, Germany.
299. Centre for Vascular Prevention, Danube-University Krems, Krems, 3500, Austria.
300. Diabetes Research Group, King Abdulaziz University, Jeddah, 21589, Saudi Arabia.
301. Genetics of Complex Traits, University of Exeter Medical School, RILD Building University of Exeter, Exeter, EX2 5DW.
302. European Centre for Environment and Human Health, University of Exeter Medical School, The Knowledge Spa, Truro, TR1 3HD, UK.
303. Department of Cardiology, University Medical Center Groningen, University of Groningen, the Netherlands.
304. Durrer Center for Cardiogenetic Research, ICIN-Netherlands Heart Institute, Utrecht, The Netherlands.
305. Center of Medical Systems Biology, Leiden, 2300 RC, The Netherlands.
306. Department of Endocrinology, University of Groningen, University Medical Center Groningen, Groningen, 9700 RB, The Netherlands.
307. Divisions of Endocrinology and Genetics and Center for Basic and Translational Obesity Research, Boston Children's Hospital, Boston MA 02115 USA.
308. Department of Biological Psychology, Vrije Universiteit, Amsterdam, the Netherlands.
309. Behavioural Science Institute, Radboud University, Nijmegen, the Netherlands.
310. School of Nutrition, Laval University, Quebec, G1V 0A6, Canada.

311. Interfaculty Institute for Genetics and Functional Genomics, University Medicine Greifswald, 17475, Germany.
312. Research Unit of Molecular Epidemiology, Helmholtz Zentrum München, German Research Center for Environmental Health, D-85764 Neuherberg, Germany.
313. Institute of Epidemiology II, Helmholtz Zentrum München - German Research Center for Environmental Health, D-85764 Neuherberg, Germany.
314. Institute of Cellular Medicine, Newcastle University, Newcastle upon Tyne, NE1 7RU, UK.
315. Centre for Population Health Sciences, Usher Institute for Population Health Sciences and Informatics, Teviot Place, Edinburgh, EH8 9AG, Scotland.
316. Department of Endocrinology, University of Groningen, University Medical Center Groningen, the Netherlands.
